# Adiponectin and leptin levels in mothers, fetuses, and neonates with intrauterine growth restriction compared to those with appropriate gestational age

**DOI:** 10.3389/fendo.2026.1827618

**Published:** 2026-05-26

**Authors:** Nuzhat Parveen, Naveed Iqbal, Abdelrahim Awadelkarim Abdelrahman Mohamed, Syed Monowar Alam Shahid, Rania Abdeen Hussain Abdalla, Gamal Eldin Mohamed Osman Elhussein, Mohammad Asim Azhar, Mohd. Saleem, Mohd Shahid Khan

**Affiliations:** 1 Department of Obstetrics and Gynecology, College of Medicine, University of Ha’il, Ha’il, Saudi Arabia; 2 Department of Biochemistry, University of Hail, Ha’il, Saudi Arabia; 3 Department of Pediatrics, College of Medicine, University of Ha’il, Ha’il, Saudi Arabia; 4 Organ Transplant Center of Excellence, King Faisal Hospital and Research Center, Riyadh, Saudi Arabia; 5 Department of Pathology, College of Medicine, University of Ha’il, Ha’il, Saudi Arabia; 6 Department of Microbiology, Hind Institute of Medical Sciences, Sitapur, Uttar Pradesh, India

**Keywords:** adiponectin, biomarkers, fetuses, intrauterine growth restriction, leptin

## Abstract

**Objective:**

Intrauterine growth restriction (IUGR) is associated with impaired fetal growth and altered metabolic regulation. Adipokines such as adiponectin and leptin play key roles in energy homeostasis and placental function. This study aimed to evaluate adiponectin and leptin levels in maternal peripheral blood, umbilical cord blood, and neonatal peripheral blood, and to investigate associated changes in placental gene expression and DNA methylation of adipokine-related genes in IUGR compared to appropriate-for-gestational-age (AGA) pregnancies.

**Materials and methods:**

This case–control study included 100 IUGR and 100 AGA full-term pregnancies. Maternal peripheral blood samples were collected at delivery, umbilical cord blood immediately after birth, and neonatal peripheral blood within 24 hours postpartum. Plasma adiponectin and leptin concentrations were measured using standardized immunoassays. Placental tissue samples were analyzed for gene expression of ADIPOQ, LEP, and WFS1, along with DNA methylation profiling using established molecular techniques. Statistical analyses were performed using appropriate tests, with p < 0.05 considered significant.

**Results:**

Adipokine levels exhibited compartment-specific variations. Adiponectin levels were significantly reduced in maternal and neonatal peripheral blood in the IUGR group, while no significant difference was observed in cord blood. Leptin levels showed differential regulation: increased levels in umbilical cord blood and decreased levels in neonatal peripheral blood in IUGR, whereas maternal levels were comparable between groups. Placental analysis revealed significant downregulation of ADIPOQ and altered expression of LEP and WFS1 in IUGR. DNA methylation analysis demonstrated increased methylation of ADIPOQ and LEP genes in placental tissue, with an inverse relationship between methylation and gene expression.

**Conclusion:**

IUGR is associated with distinct, compartment-specific alterations in adipokine profiles and significant epigenetic and transcriptional changes in placental tissue. These findings highlight the role of placental epigenetic regulation in adipokine signaling and emphasize the importance of tissue-specific mechanisms in the pathophysiology of fetal growth restriction.

## Introduction

Intrauterine growth restriction (IUGR) poses a significant challenge in both clinical and public health contexts, characterized by fetal growth that falls below the 10th percentile for gestational age. This condition is linked to heightened risks of perinatal complications, mortality, and long-term health issues, including cardiovascular diseases, insulin resistance, and metabolic syndrome (1). IUGR results from a variety of factors, with key contributors being placental dysfunction, maternal malnutrition, and impaired nutrient transfer between mother and fetus. Adipokines, especially adiponectin and leptin, have received considerable attention for their roles in fetal growth and metabolism. Adiponectin, known for its anti-inflammatory and insulin-sensitizing properties, aids in glucose and lipid metabolism, while leptin, a pro-inflammatory adipokine, plays a crucial role in energy balance and appetite control (2). The dysregulation of these adipokines in pregnancies affected by IUGR emphasizes their potential involvement in the condition’s development (3). The levels of adiponectin and leptin in mothers, fetuses, and neonates are believed to reflect the metabolic and inflammatory environment within the maternal-fetal-placental unit (4). Previous research has produced mixed results regarding adiponectin and leptin levels in IUGR pregnancies, with some studies reporting lower adiponectin and higher leptin levels, while others have observed variability across maternal, fetal, and neonatal levels. These inconsistencies highlight the necessity for further research into the specific roles and mechanisms of adipokines in IUGR (5). Adiponectin levels are typically lower in pregnancies affected by IUGR, which can negatively impact glucose metabolism and fetal development. In contrast, leptin levels present a more complex picture: both maternal and fetal leptin levels tend to be elevated, likely as a compensatory response, whereas neonatal leptin levels are often lower, suggesting potential metabolic difficulties after birth (4). These observations prompt further investigation into the regulatory mechanisms that control adipokine expression and their effects on fetal growth and neonatal adaptation.

This study aims to fill these knowledge gaps by systematically comparing adiponectin and leptin levels in maternal, fetal, and neonatal contexts between IUGR pregnancies and those that are appropriate for gestational age (AGA). Gaining insight into these differences is essential for understanding the underlying pathophysiological processes of IUGR and for identifying potential biomarkers that could aid in early diagnosis and treatment. Ultimately, this research aims to advance understanding of adipokine dynamics in IUGR pregnancies by providing a thorough evaluation of their influence on fetal growth regulation. The results may lead to innovative strategies to reduce the negative outcomes associated with IUGR, thereby improving the immediate and long-term health prospects of affected newborns.

## Materials and methods

### Study design and participants

This cross-sectional study was conducted at the University Medical Clinics and the Maternity and Children’s Hospital from January to December 2024. We recruited a total of 200 pregnant women and their newborns, dividing them into two groups: (i) the IUGR group (n=100), and (ii) the AGA group (n=100). All enrolled pregnancies were full-term (around 37–40 weeks of gestation) at delivery to minimize the potential influence of gestational age–related variations in cord blood adiponectin and leptin levels. The IUGR group was defined by ultrasonographic evidence of fetal growth falling below the 10th percentile, while the AGA group consisted of fetuses with growth rates between the 10^th^ and 90^th^ percentiles. We excluded participants with maternal metabolic disorders, multiple pregnancies, and pre-existing conditions such as diabetes and hypertension. Maternal BMI was calculated during the third trimester at the time of study enrollment using measured weight and recorded height. Pre-pregnancy BMI was not consistently available in hospital records, and therefore could not be included to avoid recall bias and incomplete datasets. Gestational weight gain was not analyzed due to insufficient availability of accurately documented longitudinal weight measurements across pregnancy.

Maternal glucose measurements were obtained during the third trimester; however, sampling was not consistently performed under fasting conditions due to clinical workflow, and therefore reflects random (non-fasting) glucose values.

### Exclusion criteria

Women with pregestational diabetes or gestational diabetes mellitus (GDM), diagnosed using the standardized oral glucose tolerance test (OGTT) at 24–28 weeks of gestation, were excluded from the study to minimize metabolic confounding.

### Sample collection

Before delivery, 5 mL of maternal venous blood was collected during the third trimester, pre- labor, into serum separator and EDTA tubes after a fast of at least 6 hours, when clinically possible. 5 ml of umbilical venous blood was collected on a clamped part of the cord under sterile conditions immediately after childbirth and before placental expulsion.

For the purpose of this study, the maternal compartment was defined as maternal peripheral blood, and the fetal compartment as neonatal peripheral blood collected at delivery. Placental tissue and umbilical cord samples were analyzed separately and are considered extra-embryonic tissues.

Cord blood samples were collected immediately after delivery, centrifuged to separate plasma, and the plasma aliquots were stored at −80 °C until analysis to maintain sample stability.

Placental tissue biopsies of about 1–2 cm³ were excised on both maternal and fetal sides within 15–30 minutes after birth, taking care to avoid infarcts and calcifications and to rinse in cold PBS to remove excess blood.

Gross placental morphological parameters, including placental weight, infarction, calcification, and vascular lesions, were not systematically recorded in the study protocol. Tissue samples were intentionally collected from macroscopically normal regions, avoiding areas with visible infarction or calcification, to preserve RNA and DNA integrity for molecular and epigenetic analyses.

Moreover, 1–2 mL of neonatal peripheral blood was collected within 24 hours of birth, before the initial full feed was possible. All specimens were properly labeled with anonymized identification codes, time-stamped, and stored under controlled conditions for later analysis.

### Biochemical analysis

Serum concentrations of adiponectin and leptin were assessed using enzyme-linked immunosorbent assay (ELISA) kits from Thermo Fisher Scientific, America, according to the manufacturer’s guidelines. Each assay batch included quality controls to guarantee accuracy and reproducibility.

### Molecular analysis

To explore the molecular regulation of adiponectin and leptin, total RNA was extracted from placental tissue samples using the QiAmp RNA Isolation Kit (Qiagen, Germany). The integrity of the RNA was evaluated using an Agilent Bioanalyzer and quantified with a Nanodrop spectrophotometer. Reverse transcription was carried out using a cDNA synthesis kit from a specified manufacturer. Quantitative real-time polymerase chain reaction (qRT-PCR) was performed using specific primers targeting ADIPOQ (adiponectin) and LEP (leptin), along with the reference genes GAPDH and β-actin for normalization. The expression levels were determined using the 2^-ΔΔCt^ method. The stability of the reference genes (GAPDH and β-actin) was confirmed by analyzing Ct value variability across all samples; both genes showed minimal intergroup variation and low coefficients of variation, supporting their suitability for normalization. Correlation analyses between DNA methylation and gene expression were performed exclusively using placental tissue samples.

### Epigenetic analysis

DNA was extracted from placental tissues using a Qiagen DNA extraction kit. The extracted DNA was subjected to bisulfite conversion using the Qiagen EpiTect Bisulfite kit following the manufacturer’s protocol. Bisulfite-converted DNA was amplified, and promoter methylation levels for ADIPOQ and LEP were quantified using pyrosequencing. Methylation levels for individual CpG sites were calculated as a percentage of methylated cytosines over the total cytosines, and the average methylation across all CpG sites in the promoter regions was determined. Pyrosequencing reactions were performed on the PyroMark Q24 system (Qiagen). Methylation levels at individual CpG sites were quantified using PyroMark Q24 software, which calculates the proportion of methylated cytosines from peak height ratios in the pyrogram. Built-in bisulfite conversion and assay controls were used to verify data quality. The average methylation percentage across analyzed CpG sites within each promoter region was used for downstream statistical analysis. The pyrosequencing assays targeted predefined CpG sites within the promoter regions and were not designed to detect sequence polymorphisms; however, no atypical pyrogram patterns suggestive of common sequence variants were observed in the analyzed regions.

### Statistical analysis

Data were analyzed using SPSS version 22 and GraphPad Prism (Version 7.0). Continuous variables were expressed as mean ± standard deviation (SD) or median [interquartile range (IQR)] based on distribution. Group comparisons were performed using Student’s t-test or Mann–Whitney U-test. Correlations between molecular parameters and biochemical levels were evaluated using Pearson or Spearman correlation coefficients. A *p*-value <0.05 was considered statistically significant.

### Ethical considerations

The study received Institutional Ethics Committee approval (H-2023-425) and was conducted in accordance with the Declaration of Helsinki. All participants provided written informed consent before enrolling in the study.

## Results

### Maternal BMI

**Figure 1:**
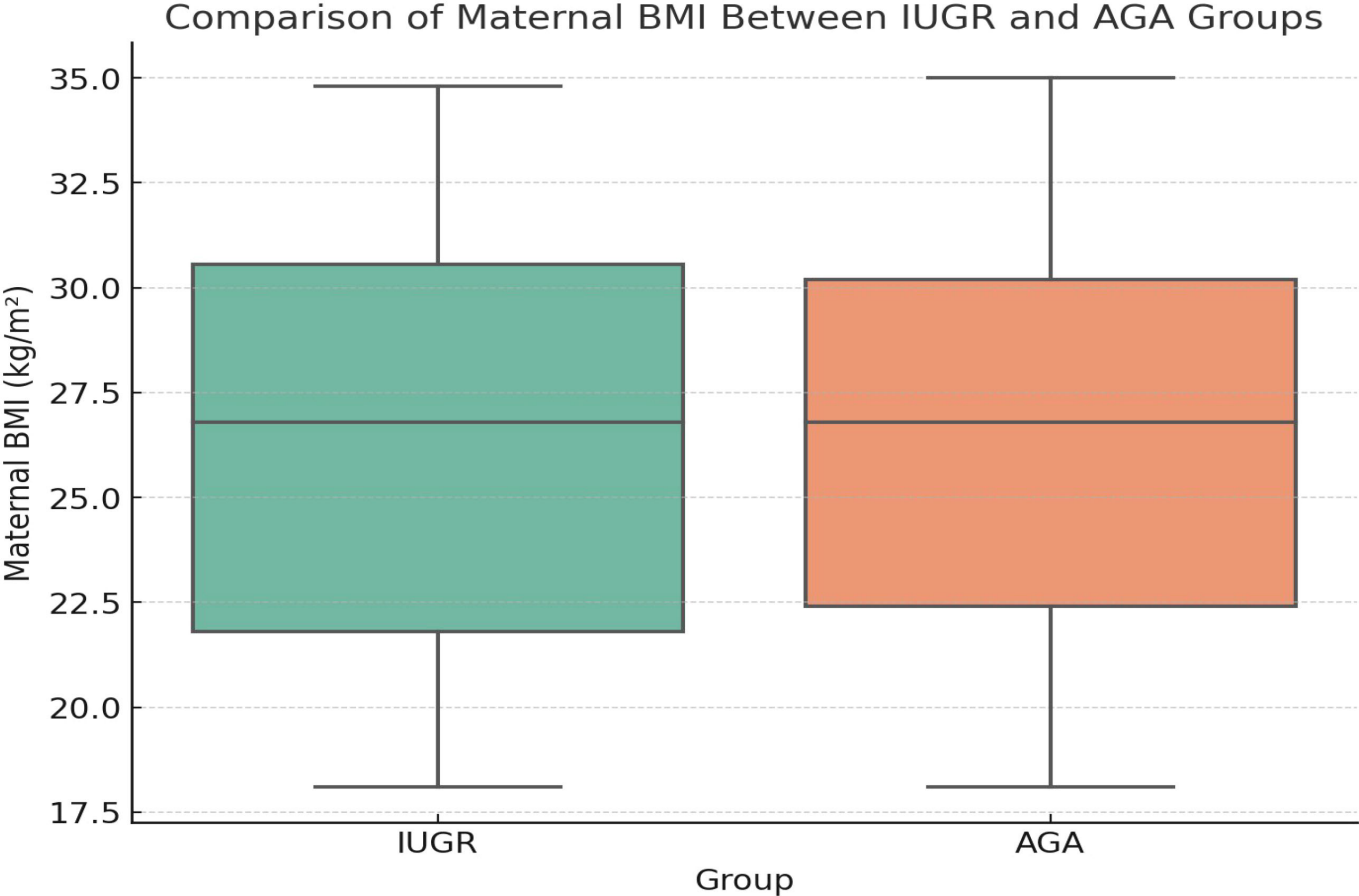
Distribution of maternal body mass index (BMI) in IUGR and AGA pregnancies. Box plots represent the distribution of maternal BMI values (kg/m2) in pregnancies complicated by IUGR (n=100) compared with AGA (n=100) pregnancies. The central line indicates the median, and the box represents the interquartile range (IQR).

**Figure 1** illustrates the distribution of maternal body mass index (BMI) in pregnancies complicated by IUGR compared with those with AGA neonates. In the IUGR group, Q1(25^th^ percentile) was found ≈ 22.0 kg/m, Q3(75^th^ percentile) was ≈ 30.5 kg/m^2^ and IQR was around 8.5 kg/m^2^. In the AGA group, Q1(25^th^ percentile) was found ≈ 22.5 kg/m, Q3(75^th^ percentile) was ≈ 30.0 kg/m^2^and IQR was around 7.5 kg/m^2^.

Mothers in the IUGR group had a higher median BMI and a broader interquartile range, indicating greater variability in maternal adiposity in growth-restricted pregnancies. In contrast, the AGA group showed slightly lower median BMI values and a narrower interquartile range, reflecting a more homogeneous BMI distribution.

The whisker ranges further indicate that BMI values are higher in the IUGR cohort than in the AGA group. Several outliers were also observed, particularly in the IUGR group, suggesting the presence of extreme BMI values among some participants. Overall, the distribution pattern presented in **Figure 1** indicates that higher maternal BMI was more frequently observed in pregnancies complicated by IUGR, suggesting a potential association between maternal metabolic status and impaired fetal growth.

Maternal BMI distributions were comparable between the IUGR and AGA groups. In the IUGR group, BMI values ranged approximately from 18 to 35 kg/m^2^, with a median of 26.5–27 kg/m^2^ and an IQR of 22-30.5 kg/m^2^. In the AGA group, BMI values showed a similar distribution, ranging approximately from 18 to 35 kg/m^2^, with a median of approximately 26.5–27 kg/m^2^ and an IQR of approximately 22.5–30 kg/m^2^. The substantial overlap in both median and IQR between the two groups indicates no clear visual difference in maternal BMI distribution.

### Maternal glucose levels

**Figure 2:**
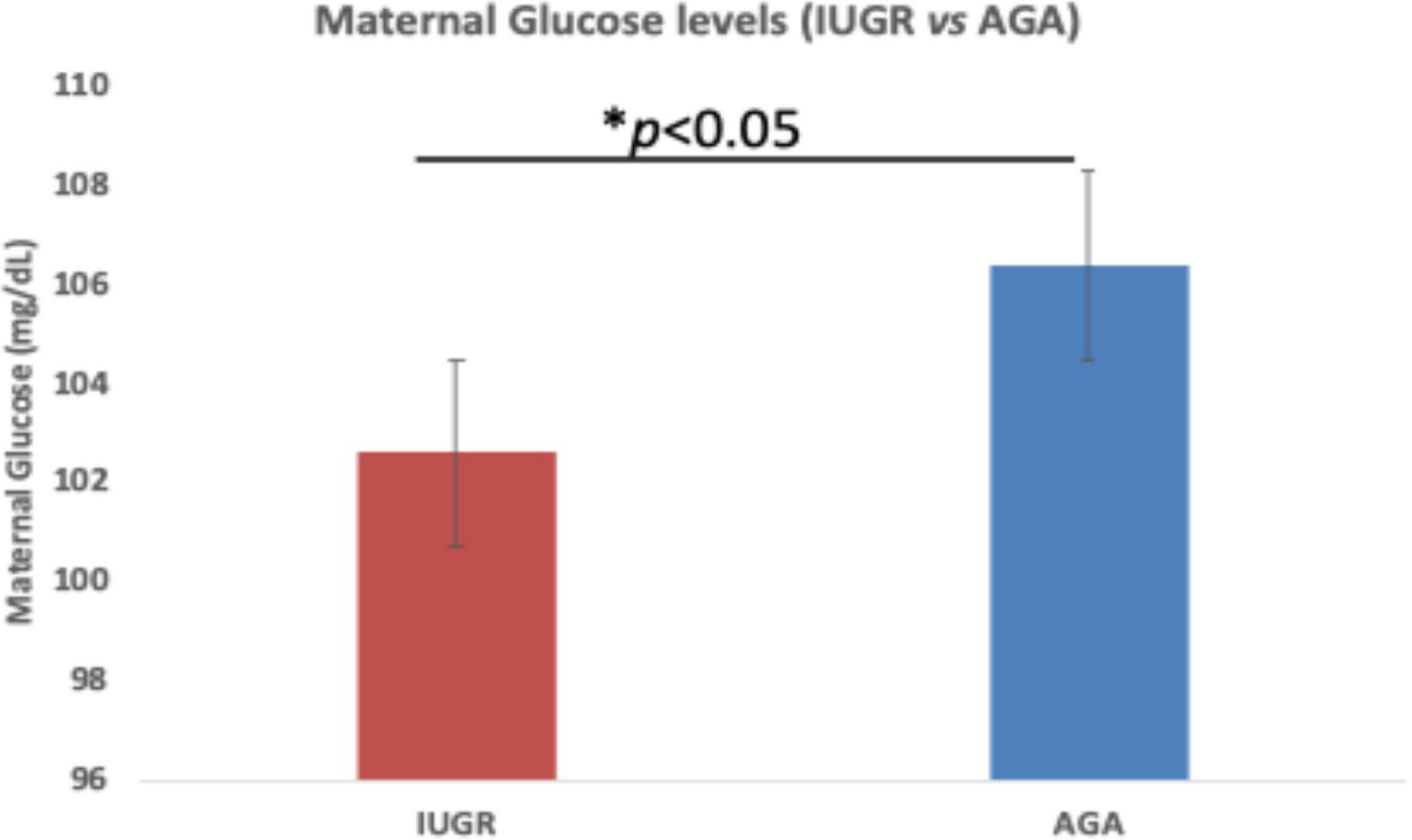
Mean maternal glucose concentrations in AGA and IUGR pregnancies. Bar graphs represent the mean maternal glucose levels (mg/dL) ± standard deviation (SD) in the IUGR group (n = 100) and AGA group (n = 100). Statistical comparisons between groups were conducted using the Student’s unpaired t-test. *p < 0.005.

The bar graph (**Figure 2**) shows that, on average, maternal glucose levels are slightly lower in the IUGR group (102.6 mg/dL) than in the AGA group (106.4 mg/dL), though the difference is quite small (**Figure 2**).

### Maternal triglycerides

**Figure 3:**
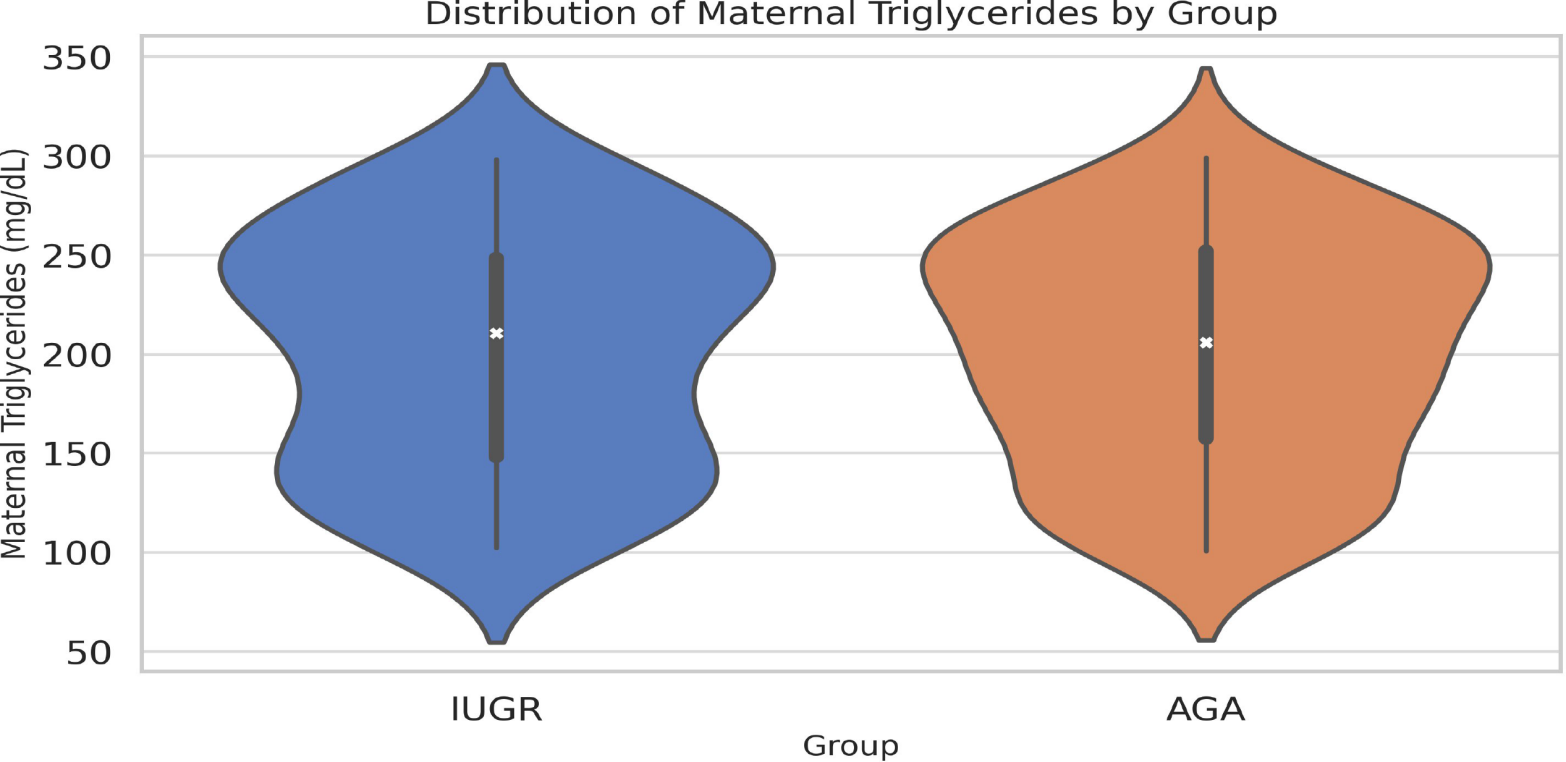
Triglyceride level of mothers in IUGR and AGA groups. Violin plots of the distribution of maternal triglyceride concentrations (mg/dL) in pregnancies complicated by IUGR (n=100) and AGA, (n=100) pregnancies. The width of each violin indicates the distribution of the data, and the line in the middle of each violin shows the median. Data were compared statistically between the groups by Student’s unpaired t-test. Significant differences were defined as *p* < 0.05.

The violin plot in **Figure 3** shows the distributions of maternal triglyceride concentrations in the IUGR and AGA groups, with distributions largely overlapping. In the IUGR group, triglyceride levels appear to range from approximately 100 to 300 mg/dL, with a median of 210–220 mg/dL. The distribution shows moderate density in the ranges of 130–160 mg/dL and 230–260 mg/dL, indicating a bimodal tendency. In the AGA group, triglyceride levels span a similar range, approximately 100–300 mg/dL, with a median around 210–220 mg/dL. The density distribution is relatively broader between 150–270 mg/dL, without a sharply defined peak. Overall, both groups demonstrate comparable central tendency and spread, with substantial overlap in interquartile ranges and distribution density, suggesting no clear visual difference in triglyceride profiles between IUGR and AGA pregnancies.

### Maternal adiponectin and leptin levels

**Figure 4:**
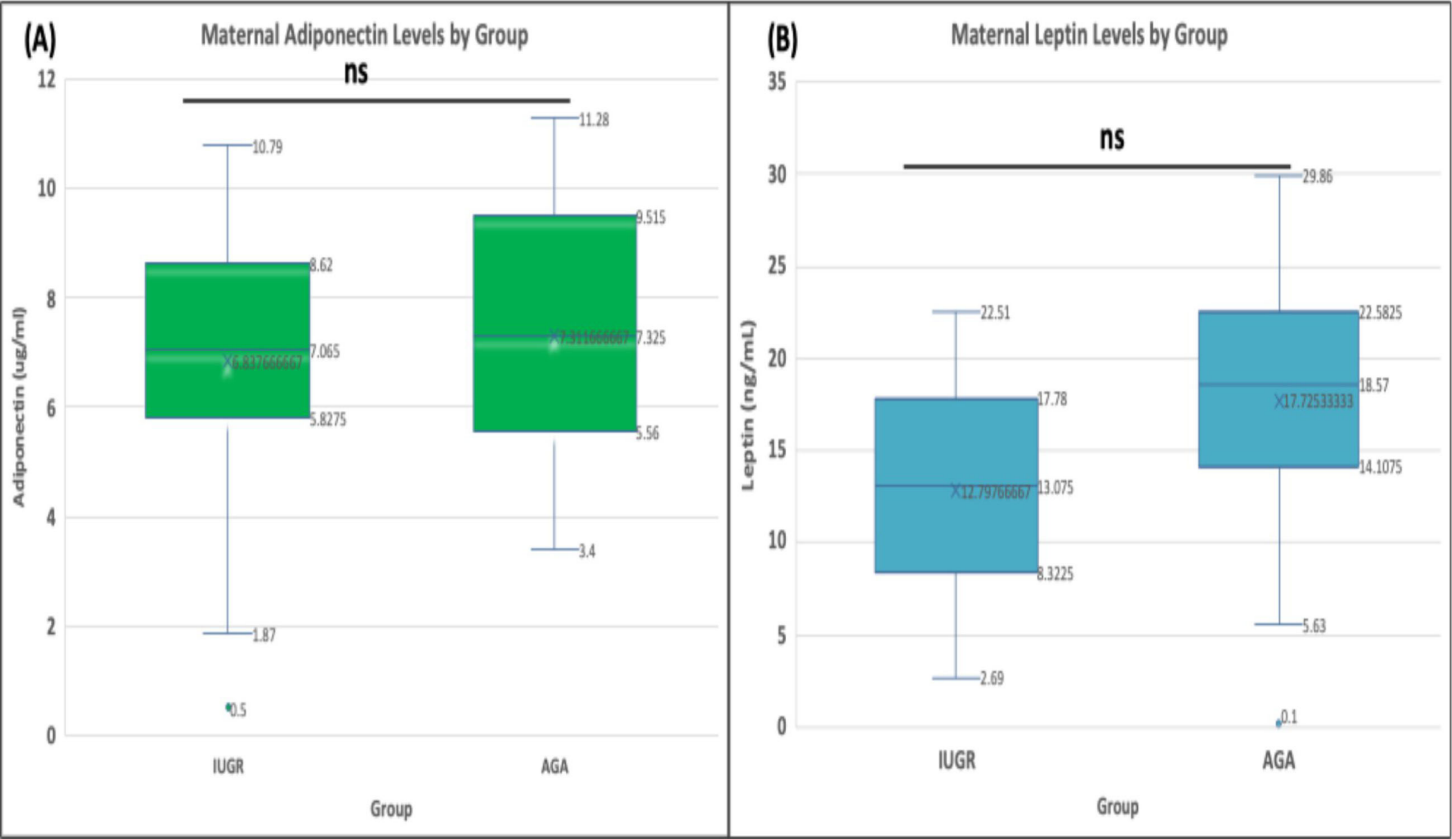
Maternal adipokine profiles in IUGR and AGA pregnancies. **(A)** Maternal adiponectin levels (µg/mL) and **(B)** maternal leptin levels (ng/mL) in pregnancies complicated by IUGR (n=100) and AGA (n=100) pregnancies. Box plots represent the interquartile range (25^th^-75^th^ percentile), horizontal lines indicate the median, “X” denotes the mean, whiskers represent minimum and maximum values, and dots indicate outliers. Statistical comparisons between groups were performed using the Student’s unpaired t-test. ns, not significant.

No statistically significant differences in maternal adiponectin and leptin levels were observed between the IUGR and AGA groups (*p*>0.05). **Figure 4A** illustrates maternal adiponectin concentrations in the IUGR and AGA groups using box-plot distribution analysis. In the IUGR group, adiponectin levels ranged from 1.87 to 10.79 µg/mL, with a median of 7.065 µg/mL and a mean value of 6.84 µg/mL, indicating moderate dispersion around the central tendency. The IQR ranged from 5.8275 µg/mL (Q1) to 8.62 µg/mL (Q3), indicating relatively compact variability across most subjects, although a low outlier at 0.5 µg/mL was observed. In comparison, the AGA group demonstrated slightly higher adiponectin levels, ranging from 3.4 to 11.28 µg/mL, with a median of 7.325 µg/mL and a mean of 7.32 µg/mL. The AGA interquartile distribution (Q1 = 5.56 µg/mL, Q3 = 9.515 µg/mL) shows a broader upper spread, reflecting increased adiponectin concentrations relative to IUGR pregnancies. Although maternal adiponectin levels were numerically higher in the AGA group compared with the IUGR group, this difference was not statistically significant (Student’s unpaired t-test, *p* = 0.18).

**Figure 4B** presents maternal leptin concentrations comparing IUGR and AGA pregnancies. In the IUGR group, leptin levels ranged from 2.69 to 22.51 ng/mL, with a median of 13.075 ng/mL and a mean of 12.80 ng/mL, indicating moderate variability across subjects. The interquartile range (Q1 = 8.3225 ng/mL, Q3 = 17.78 ng/mL) indicates a relatively lower leptin concentration distribution in IUGR mothers. Conversely, the AGA group exhibited substantially higher leptin levels, ranging from 5.63 to 29.86 ng/mL, with a median of 18.57 ng/mL and a mean of 17.73 ng/mL. The wider IQR (Q1 = 14.1075 ng/mL, Q3 = 22.5825 ng/mL) demonstrates an upward shift in leptin distribution, consistent with enhanced maternal adipose signaling and placental endocrine activity. Maternal leptin levels were higher in the AGA group compared with the IUGR group; however, this difference did not reach statistical significance (Student’s unpaired t-test, *p* = 0.27).

### Neonatal peripheral adiponectin and leptin levels

**Figure 5:**
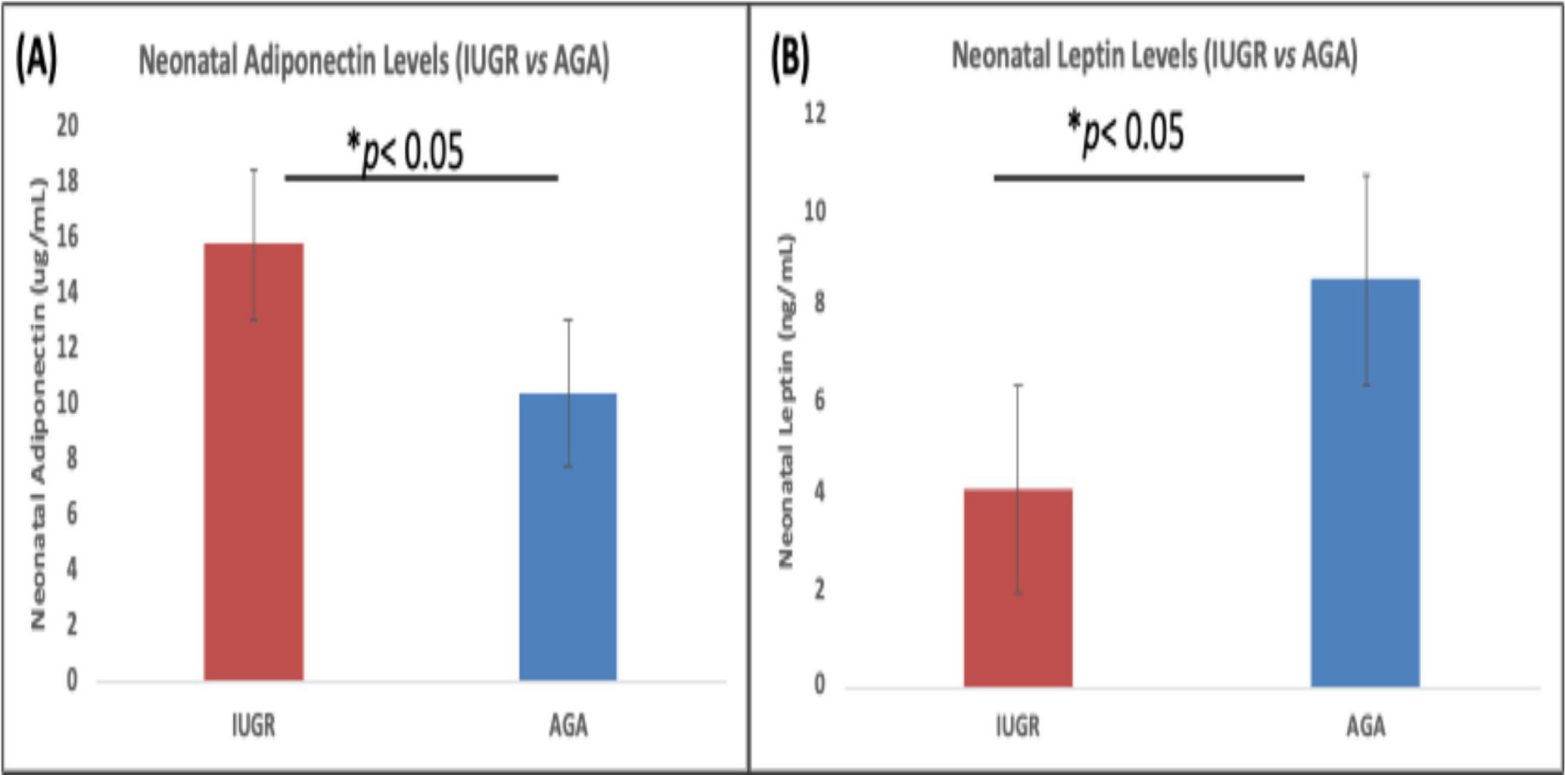
Neonatal adipokine concentrations in IUGR and AGA groups. **(A)** Neonatal adiponectin levels (µg/mL) show significantly higher concentrations in IUGR neonates compared with AGA neonates (15.8 ± 4.2 vs 10.4 ± 8.6 µg/mL; Student’s unpaired t-test, *p* < 0.005). **(B)** Neonatal leptin levels (ng/mL) show significantly lower concentrations in IUGR neonates compared with AGA neonates (4.2 ± 1.5 vs 8.6 ± 1.7 ng/mL; Student’s unpaired t-test, *p < 0.005). Data are presented as mean ± standard deviation (SD).

Neonatal peripheral blood analysis demonstrated significant differences in adipokine concentrations between IUGR and AGA groups. Neonatal adiponectin levels were significantly higher in the IUGR group (15.8 ± 4.2 µg/mL) compared with the AGA group (10.4 ± 8.6 µg/mL) (Student’s unpaired t-test, *p* < 0.005) (**Figure 5A**). In contrast, neonatal leptin concentrations were significantly lower in the IUGR group (4.2 ± 1.5 ng/mL) compared with the AGA group (8.6 ± 1.7 ng/mL) (Student’s unpaired t-test, *p* < 0.005) (**Figure 5B**).

These findings indicate a divergent pattern of adipokine regulation in IUGR neonates, characterized by elevated adiponectin and reduced leptin levels in the early postnatal period.

### Cord adiponectin and cord leptin levels

**Figure 6:**
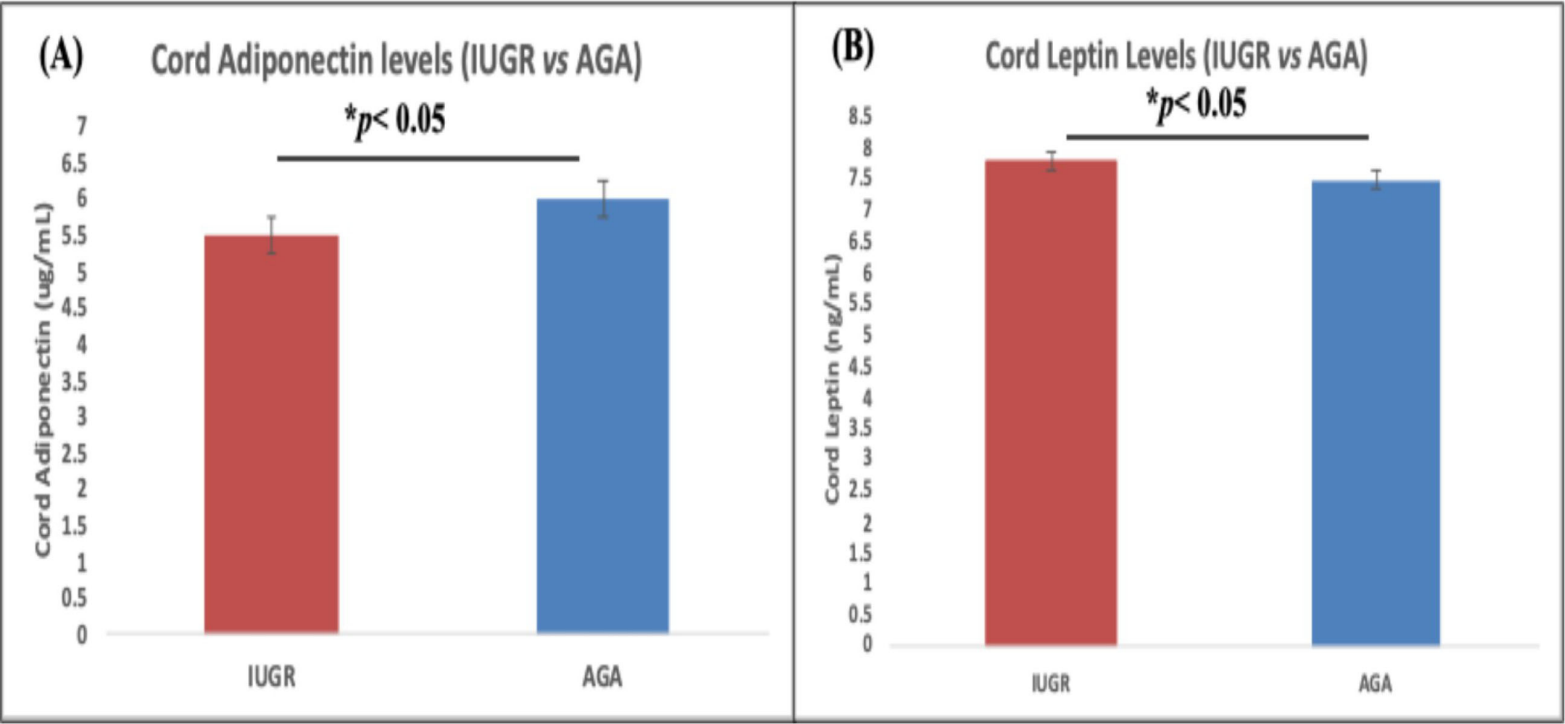
Cord blood adipokine concentrations in IUGR and AGA pregnancies. Cord adiponectin (µg/mL) and leptin (ng/mL) concentrations are shown for AGA and IUGR groups. Bars represent mean values, and error bars indicate variability. Cord adiponectin levels were significantly lower in the IUGR group compared with AGA **(A)**, while cord leptin levels were significantly higher in the IUGR **(B)**. Statistical comparisons were performed using the Student’s unpaired t-test (*p < 0.005).

Cord blood adiponectin concentrations were significantly lower in the IUGR group (~5.5 µg/mL) compared with the AGA group (~6.0 µg/mL), as determined by the Student’s unpaired t-test (*p* < 0.005) (**Figure 6A**). This reduction indicates impaired fetal adiponectin regulation in pregnancies complicated by intrauterine growth restriction. In contrast, cord blood leptin concentrations were slightly significantly higher in the IUGR group (~7.8 ng/mL) compared with the AGA group (~7.5 ng/mL) (Student’s unpaired t-test, *p* < 0.005) (**Figure 6B**). This increase may reflect a compensatory response to intrauterine stress or altered placental leptin production in growth-restricted fetuses. Collectively, these findings demonstrate a differential pattern of adipokine regulation in IUGR, characterized by reduced adiponectin and increased leptin levels in the fetal compartment.

Placental gene expression analysis revealed significant differences in the transcriptional levels of ADIPOQ and LEP between pregnancies complicated by IUGR and those with AGA neonates. The relative expression of ADIPOQ was markedly reduced in the IUGR group, with a mean expression level of 0.38 ± 0.12, compared with 0.86 ± 0.21 in the AGA group (**Table 1**). This represents a substantial decrease in adiponectin gene transcription in placentas associated with growth-restricted pregnancies. The difference between the two groups was statistically highly significant (*p* < 0.001), indicating a strong association between IUGR and reduced ADIPOQ expression.

**Table 1:** Placental gene expression levels in IUGR and AGA groups.

Gene	Group	Relative expression (mean ± SD)	*p-*value
ADIPOQ	IUGR	0.38 ± 0.12	<0.001
	AGA	0.86 ± 0.21	
LEP	IUGR	0.31 ± 0.10	<0.001
	AGA	0.74 ± 0.18	

Placental tissue collected at delivery was used for all gene expression analysis.

A similar trend was observed for the LEP gene. Placental LEP expression was significantly lower in the IUGR group, with a mean relative expression of 0.31 ± 0.10, whereas the AGA group demonstrated a higher expression level of 0.74 ± 0.18. The reduction in leptin gene transcription in IUGR placentas was also statistically significant (*p* < 0.001) (**Table 1**). Collectively, these findings indicate that both ADIPOQ and LEP genes exhibit significantly reduced expression in placental tissues from IUGR pregnancies, supporting the hypothesis that dysregulation of adipokine-related genes may contribute to the altered metabolic and endocrine environment associated with impaired fetal growth.

To further explore the regulatory mechanisms underlying these expression changes, DNA methylation analysis was subsequently performed.

### Epigenetic studies

In the present study, methylation levels at the ADIPOQ (adiponectin) and LEP (leptin) gene promoters were analyzed to assess potential epigenetic regulation in placental tissues from pregnancies affected by IUGR, compared with those with AGA (**Table 2**). The analysis revealed a significant increase in the methylation percentage of the ADIPOQ promoter in the IUGR group (65.2 ± 7.8%) compared to the AGA group (45.1 ± 7.5%, *p* < 0.001). Similarly, the LEP promoter demonstrated a higher methylation percentage in the IUGR group (70.3 ± 7.6%) than in the AGA group (50.2 ± 7.9%, *p* < 0.001). These observations are consistent with the hypothesis that epigenetic modifications, such as DNA methylation, could play a critical role in the dysregulation of adipokine genes in IUGR pregnancies.

**Table 2:** Comparison of gene methylation levels between IUGR and AGA groups.

Gene	Group	Mean methylation (%)	Standard deviation (SD)	*p*-value
ADIPOQ	IUGR	65.2	7.8	<0.001
	AGA	45.1	7.5	
LEP	IUGR	70.3	7.6	<0.001
	AGA	50.2	7.9	

Placental tissue collected at delivery was used for DNA methylation analyses.

Hypermethylation of promoter regions is typically associated with reduced gene transcription due to inhibition of transcription factor binding or the recruitment of methyl-binding proteins that promote chromatin condensation. The observed hypermethylation of the ADIPOQ promoter in IUGR may explain the reduced expression levels of adiponectin, a hormone essential for regulating fetal growth, energy metabolism, and placental function. Similarly, increased methylation of the LEP promoter may contribute to leptin dysregulation, which plays a pivotal role in fetal growth, angiogenesis, and placental nutrient transport. These findings suggest that aberrant epigenetic modifications in the placental tissue could disrupt the normal expression of adipokines, contributing to the pathophysiology of IUGR.

These results highlight the importance of examining epigenetic changes, as they provide insights into the molecular mechanisms underlying pregnancy complications such as IUGR. They also open avenues for further investigation into whether these methylation changes are reversible and whether they can serve as potential biomarkers for early detection or therapeutic targets for intervention. By linking epigenetic modifications to the altered gene expression observed in IUGR, this study underscores the complex interplay between genetics and epigenetics in placental development and fetal growth, contributing to the growing body of evidence on the molecular basis of pregnancy disorders.

**Table 2** presents data comparing the mean methylation levels of the two genes ADIPOQ and LEP between infants with IUGR and those with AGA. The methylation percentages for both genes are significantly higher in the IUGR group compared to the AGA group, as indicated by the *p*-value (<0.001) (**Table 2**). This suggests a potential link between altered gene methylation patterns and IUGR, with implications for understanding epigenetic regulation in this condition.

**Figure 7:**
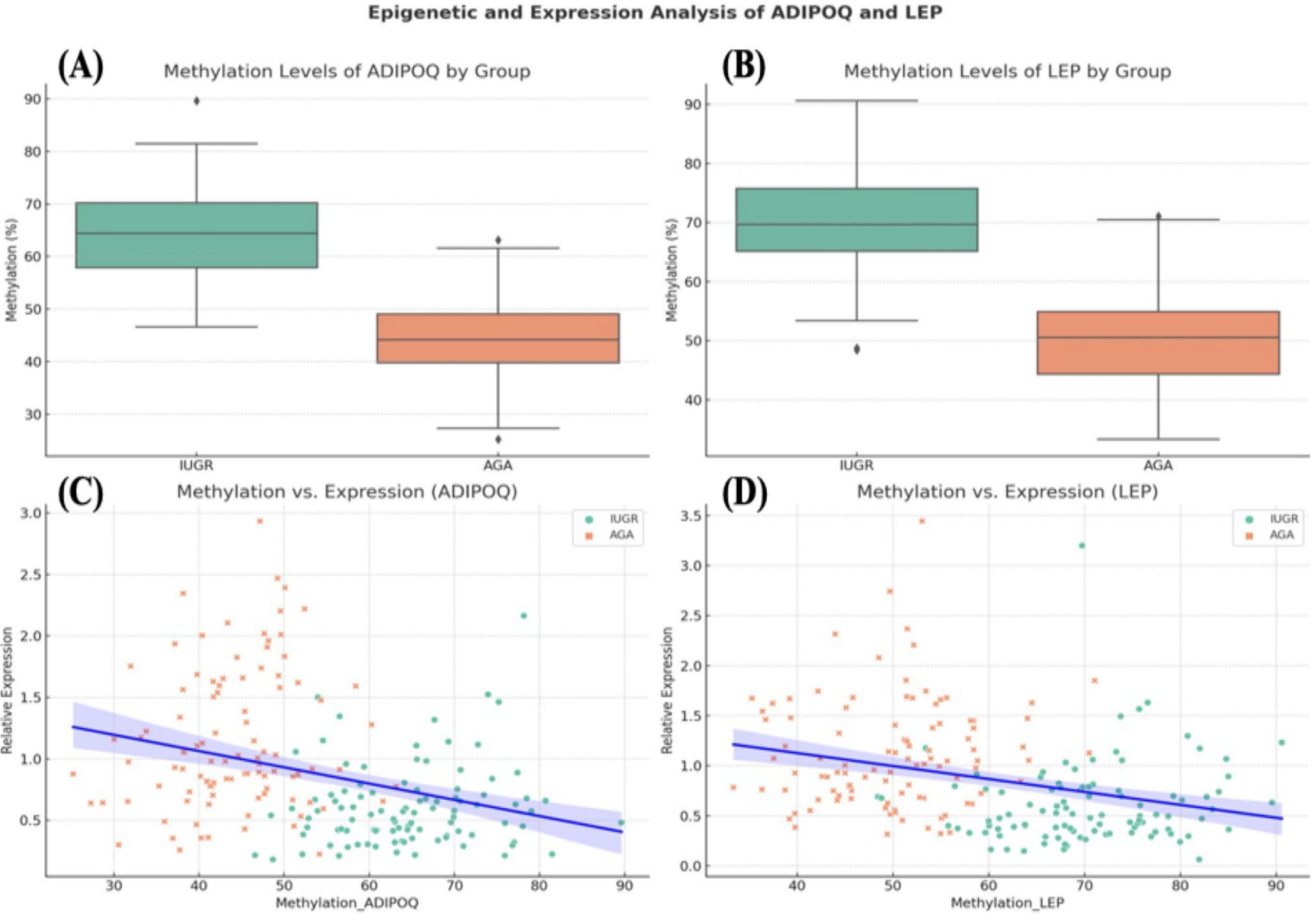
Analysis of epigenetics and expression for ADIPOQ and LEP in IUGR and AGA groups. **(A)** A comparison of ADIPOQ methylation levels between the IUGR and AGA groups, **(B)** a comparison of LEP methylation levels between the IUGR and AGA groups, **(C)** the relationship between ADIPOQ methylation levels and relative gene expression, categorized by group (IUGR and AGA), and **(D)** the relationship between LEP methylation levels and relative gene expression, categorized by group (IUGR and AGA).

The boxplot illustrates the distribution of ADIPOQ methylation levels in the IUGR and AGA groups (**Figure 7**). The boxplot illustrates the distribution of ADIPOQ methylation levels in IUGR and AGA groups. In the IUGR group, methylation values range approximately from 50% to 80%, with a median around 65% and an IQR spanning roughly 58% to 72%. In contrast, the AGA group shows lower methylation levels, ranging approximately from 30% to 65%, with a median of 48–50% and an IQR of 40%-55% (**Figure 7A**). The IUGR group shows a higher central tendency and a slightly broader distribution than the AGA group.

The distribution of LEP methylation levels shows a similar trend between groups. In the IUGR group, values range approximately from 55% to 82–83%, with a median around 72% and an IQR of approximately 65% to 77–78%. In the AGA group, methylation levels range from approximately 35% to 70%, with a median of 52–53% and an IQR of approximately 45%-60% (**Figure 7B**). Overall, the IUGR group shows higher methylation levels and a slightly broader distribution than the AGA group.

The scatterplot demonstrates an inverse relationship between ADIPOQ methylation and gene expression. As methylation increases from approximately 45% to 80%, expression levels decline from roughly 2.2–2.4-fold to approximately 0.4–0.5-fold (**Figure 7C**). The regression line indicates a clear negative trend across both IUGR and AGA groups. Data points from the IUGR group tend to cluster at higher methylation and lower expression levels. In contrast, AGA samples are more frequently distributed at lower methylation and relatively higher expression, supporting an epigenetic suppression pattern. In placental tissue samples, DNA methylation levels were inversely correlated with gene expression for both ADIPOQ and LEP.

A similar inverse association is observed between LEP methylation and gene expression levels. Methylation values ranging from approximately 50% to 80% correspond to a decrease in expression from about 2.2–2.4-fold to approximately 0.4–0.5-fold (**Figure 7D**). The regression trendline demonstrates a clear negative slope, indicating that increased methylation is associated with reduced gene expression. As observed for ADIPOQ, IUGR samples are predominantly located in the high–methylation, low–expression region. In contrast, AGA samples show a broader distribution toward lower methylation and higher expression.

## Discussions

In this study, analyses were performed on maternal serum, neonatal peripheral blood, and extra-embryonic tissues, including placenta and umbilical cord. The observed alterations in adipokine levels were primarily observed in neonatal peripheral blood and in placental and umbilical cord tissues, highlighting compartment-specific differences rather than a generalized neonatal distribution.

In contrast, maternal circulating levels did not differ significantly between the two groups. These observations suggest that dysregulation of adipokine signaling in IUGR is primarily associated with fetal and placental metabolic adaptations rather than maternal systemic changes. This distinction highlights the importance of placental and fetal endocrine regulation in the pathophysiology of growth restriction.

Maternal BMI did not differ significantly between groups, suggesting that the observed differences in fetal adipokine profiles are unlikely to be solely driven by maternal anthropometric factors. Cord blood adipokine analysis demonstrated a distinct pattern of metabolic dysregulation in IUGR pregnancies, characterized by significantly reduced adiponectin and elevated leptin levels compared with AGA controls. The decrease in cord adiponectin suggests impaired insulin-sensitizing capacity and altered energy homeostasis within the fetal compartment, potentially reflecting compromised placental nutrient transfer and metabolic signaling. In contrast, the increase in cord leptin may represent a compensatory response to intrauterine stress, possibly driven by hypoxia, placental dysfunction, or altered fetal endocrine activity. Given that both placental tissue and fetal adipocytes produce leptin, its elevation in cord blood may reflect adaptive mechanisms that regulate fetal growth under nutrient-restricted conditions. Together, these findings support the presence of early metabolic alterations in the fetal–placental unit in IUGR, which may contribute to long-term metabolic programming. In the neonatal period, adipokine profiles showed a reversal of the cord blood pattern, with significantly higher adiponectin and lower leptin levels in IUGR neonates compared with AGA counterparts. The elevation of adiponectin following birth may reflect a compensatory metabolic adaptation, potentially enhancing insulin sensitivity and facilitating energy utilization in growth-restricted infants. Conversely, the reduction in neonatal leptin is likely attributable to reduced fat mass and diminished adipose tissue development, which are characteristic features of IUGR neonates. The divergence between cord and neonatal leptin levels further suggests a transition from placental to autonomous metabolic regulation after birth, highlighting the dynamic nature of adipokine signaling during early life (3). These findings underscore the importance of postnatal metabolic reprogramming in IUGR, which may influence susceptibility to future cardiometabolic disorders. Adiponectin and leptin are closely linked to insulin signaling pathways and play critical roles in regulating glucose homeostasis during pregnancy. Although direct measurements of insulin resistance indices such as HOMA-IR or glycated hemoglobin (HbA1c) were not available in the present study, the observed reduction in fetal and neonatal adiponectin levels in IUGR pregnancies may reflect impaired insulin sensitivity within the fetal–placental unit (6). Previous studies have demonstrated that lower adiponectin concentrations are associated with reduced insulin sensitivity and altered metabolic programming, which may contribute to long-term cardiometabolic risk in growth-restricted offspring (7, 8). Similarly, leptin interacts with insulin and insulin-like growth factor (IGF) signaling pathways, further supporting the relevance of adipokine imbalance in metabolic dysregulation associated with IUGR (7, 9).

Similarly, leptin concentrations demonstrated compartment-specific variation across maternal, fetal, and neonatal compartments. In the present study, cord blood leptin levels were significantly higher in the IUGR group. In contrast, neonatal leptin levels were significantly reduced, indicating a dynamic shift in leptin regulation between intrauterine and early postnatal environments. The elevated cord leptin levels in IUGR may reflect a compensatory response to intrauterine stress, altered placental leptin production, or hypoxic signaling within the fetal–placental unit. In contrast, reduced neonatal leptin levels likely reflect diminished fat mass and impaired postnatal metabolic adaptation. These findings highlight the complex and stage-dependent role of leptin in fetal growth and metabolic programming.

The gene expression analysis performed in this study further supports the role of placental molecular regulation in IUGR, demonstrating significant alterations in adipokine-related gene expression in placental tissues from IUGR pregnancies compared with AGA controls (9, 10). Changes in placental gene expression may influence the synthesis, secretion, and signaling of adipokines within the maternal–fetal interface. These findings highlight the importance of transcriptional regulation and epigenetic modulation in the pathophysiology of fetal growth restriction, suggesting that altered gene expression may contribute to disrupted endocrine and metabolic signaling in the placenta.

Although placental morphological features were not quantitatively assessed in the present study, structural abnormalities such as reduced placental weight, infarction, and vascular lesions are well-documented in IUGR. They are closely associated with impaired placental perfusion and endocrine function (11). The molecular and epigenetic alterations observed in ADIPOQ and LEP in our study may reflect underlying structural and functional placental abnormalities, even in the absence of direct morphometric evaluation. The observed inverse correlation between DNA methylation and gene expression in placental tissue suggests epigenetic regulation of adipokine genes in IUGR.

In addition to adipokine-related genes, growing evidence indicates that genetic regulators of fetal growth may also contribute to the development of growth restriction. For example, altered expression of the C14orf132 gene has been associated with low birth weight, indicating that transcriptional regulation of specific genes may influence fetal growth trajectories (12, 13). These findings emphasize that IUGR is a multifactorial condition involving complex genetic and molecular mechanisms, including interactions among metabolic hormones, gene expression networks, and placental function.

Immune and inflammatory pathways may also contribute to impaired fetal growth. Studies have demonstrated that innate immune mediators, such as S100A8/A9, play important roles in inflammatory signaling at the maternal–fetal interface, and dysregulation of these pathways has been associated with pregnancy complications, including miscarriage and impaired fetal development (14). Inflammatory activation within the placenta may alter trophoblast function and vascular development, potentially compromising nutrient delivery to the fetus (15, 16). These mechanisms highlight the interplay between immune regulation, placental biology, and fetal growth outcomes.

Another important genetic regulator of fetal growth is the WFS1 gene, which has been implicated in glucose metabolism, insulin secretion, and the regulation of the insulin-like growth factor signaling pathway. These metabolic pathways are closely linked to fetal growth and placental nutrient transport (17, 18). Furthermore, WFS1 has been associated with mitochondrial energetics and cellular energy homeostasis, suggesting that alterations in mitochondrial function could affect placental metabolism and fetal development (19, 20). Given the established interaction between leptin signaling and metabolic pathways, WFS1 may represent an important molecular link connecting energy metabolism, endocrine regulation, and fetal growth (21).

WFS1 encodes Wolframin, an endoplasmic reticulum (ER)-associated protein that helps maintain ER homeostasis and regulates cellular stress responses. Alterations in WFS1 expression have been linked to increased ER stress and impaired insulin signaling pathways. Given that leptin signaling is sensitive to metabolic and ER stress, dysregulation of WFS1 may indirectly influence leptin production and signaling *via* ER stress–mediated mechanisms (22). This interaction may contribute to altered adipokine profiles observed in IUGR. The observed changes in WFS1 expression and methylation in placental tissue represent a potential mechanistic link to the altered leptin dynamics identified across compartments in this study.

Beyond their metabolic roles, adipokines such as leptin and adiponectin have also been implicated in neuroendocrine signaling pathways involved in emotional and behavioral regulation. Metabolic peptides interact with central nervous system pathways that influence stress responses and emotional regulation. For example, neuropeptides such as cholecystokinin (CCK) have been shown to modulate emotional responses and anxiety-related behaviors (23). Although the present study primarily focused on metabolic and growth-related mechanisms, these observations suggest that metabolic peptides may also interact with neuroendocrine pathways influencing maternal physiological adaptation during pregnancy (24, 25).

From a methodological perspective, the present study minimized potential confounding by including only full-term pregnancies and by standardizing sample collection and processing procedures. These procedures ensured the stability and reliability of adipokine measurements (26). Although absolute adipokine concentrations may vary across studies due to differences in assay methodologies and population characteristics, all samples in this study were analyzed under identical experimental conditions, supporting the internal validity of the comparisons between IUGR and AGA groups (27).

Overall, the findings of this study support the concept that fetal growth restriction is associated with dysregulation of adipokine signaling within the fetal–placental unit, rather than significant alterations in maternal circulating adipokine levels (28, 29). Reduced adiponectin and leptin concentrations in fetal and neonatal compartments, together with altered placental gene expression, suggest that placental endocrine and metabolic dysfunction may play a central role in the pathogenesis of IUGR (3, 5, 30). Future studies that integrate genetic, epigenetic, metabolic, and immunological mechanisms may further elucidate the complex molecular networks regulating fetal growth. They may help identify potential biomarkers or therapeutic targets for the early detection and management of growth-restricted pregnancies.

## Conclusion

The present study demonstrates that pregnancies complicated by IUGR are associated with significant alterations in adipokine signaling within the fetal–placental unit. Specifically, adiponectin and leptin concentrations were significantly lower in cord blood and neonatal peripheral blood in the IUGR group than in the AGA group. In contrast, maternal circulating levels did not differ significantly between the groups. These findings suggest that the metabolic dysregulation associated with IUGR is primarily localized to the placental and fetal compartments rather than the maternal systemic circulation. In addition, the observed alterations in placental gene expression related to adipokine signaling further support the role of transcriptional regulation in the pathophysiology of fetal growth restriction.

Collectively, these results highlight the importance of placental endocrine and metabolic pathways in regulating fetal growth, suggesting that dysregulation of adipokine-related signaling may contribute to impaired nutrient transport, altered metabolic adaptation, and restricted fetal development. The integration of metabolic findings with emerging evidence from genetic, epigenetic, and inflammatory pathways emphasizes the multifactorial nature of IUGR. Future research incorporating larger cohorts and multi-omics approaches may further clarify the molecular mechanisms linking adipokine signaling, placental function, and fetal growth outcomes. It may help identify potential biomarkers for the early detection and management of growth-restricted pregnancies.

### Limitations of the study

Several limitations of the present study should be considered when interpreting the findings. First, the sample size was relatively limited, which may restrict the generalizability of the results to broader populations. In addition, the study focused on selected adipokines and specific gene expression markers, whereas a complex interplay of metabolic, genetic, and inflammatory pathways governs fetal growth regulation. The absence of stratified analyses based on maternal BMI categories further limits the ability to assess the influence of maternal metabolic status on adipokine regulation.

Second, the observational, cross-sectional design of the study precludes establishing causal relationships between altered adipokine levels, gene expression changes, and the development of intrauterine growth restriction. Furthermore, although standardized protocols were followed, variability in biological conditions and assay methodologies may influence measured adipokine concentrations. The lack of comprehensive metabolic profiling, including insulin resistance parameters such as fasting insulin, HOMA-IR, and glycated hemoglobin (HbA1c), also limits the interpretation of the metabolic context of adipokine alterations. Finally, the study did not include a detailed assessment of placental morphology, such as placental weight or histopathological features, which could provide important structural correlates to the observed molecular findings. Additionally, while placental gene expression and DNA methylation were analyzed, direct quantification of adipokine protein levels in placental tissue was not performed. Future studies integrating larger cohorts, longitudinal designs, detailed metabolic profiling, and combined molecular and histopathological analyses are warranted to provide a more comprehensive understanding of the mechanisms underlying IUGR.

## Data Availability

The original contributions presented in the study are included in the article/**Supplementary Material**. Further inquiries can be directed to the corresponding author.
