# Supplementary material for: Adiponectin and leptin levels in mothers, fetuses, and neonates with intrauterine growth restriction compared to those with appropriate gestational age
Source: Front Endocrinol (Lausanne). 2026 May 26;17:1827618. doi: 10.3389/fendo.2026.1827618 (PMC13246382; doi:10.3389/fendo.2026.1827618)
Supplement: Supplementary file 1 [file Table1.docx]

| **Patient_ID**  **Supplementary Table 1:** qRT-PCR–Based Expression Profile of *ADIPOQ* and *LEP* Genes in IUGR Placental Samples (ΔCt and Relative Expression Values) | **Group** | **DeltaCt_ADIPOQ** | **DeltaCt_LEP** | **Relative_Expression_ADIPOQ** | **Relative_Expression_LEP** |
| --- | --- | --- | --- | --- | --- |
| IUGR_1 | IUGR | 5.897371322 | 6.286229888 | 0.384344612 | 0.435046708 |
| IUGR_2 | IUGR | 5.389388559 | 6.448627621 | 0.54656126 | 0.38873111 |
| IUGR_3 | IUGR | 6.01815083 | 6.866440995 | 0.353478124 | 0.290987907 |
| IUGR_4 | IUGR | 6.718423885 | 6.843041642 | 0.21755013 | 0.295745978 |
| IUGR_5 | IUGR | 5.3126773 | 4.897864506 | 0.57640964 | 1.138873565 |
| IUGR_6 | IUGR | 5.312690434 | 5.249739968 | 0.576404393 | 0.892381264 |
| IUGR_7 | IUGR | 6.763370252 | 6.412028214 | 0.210876965 | 0.398718896 |
| IUGR_8 | IUGR | 6.113947783 | 6.411028761 | 0.330768989 | 0.398995211 |
| IUGR_9 | IUGR | 5.124420491 | 6.412038149 | 0.656753177 | 0.39871615 |
| IUGR_10 | IUGR | 5.934048035 | 9.082185193 | 0.374696819 | 0.062642557 |
| IUGR_11 | IUGR | 5.129265846 | 6.456712409 | 0.654551142 | 0.386558774 |
| IUGR_12 | IUGR | 5.127416197 | 6.908452512 | 0.655390866 | 0.282636479 |
| IUGR_13 | IUGR | 5.693569817 | 6.763201411 | 0.4426609 | 0.312574217 |
| IUGR_14 | IUGR | 3.969375804 | 6.521113001 | 1.46253167 | 0.369682613 |
| IUGR_15 | IUGR | 4.120065734 | 5.747784604 | 1.317477146 | 0.631864664 |
| IUGR_16 | IUGR | 5.050169977 | 6.607175376 | 0.691438908 | 0.348274493 |
| IUGR_17 | IUGR | 4.689735104 | 5.381739828 | 0.887678134 | 0.814356423 |
| IUGR_18 | IUGR | 5.751397866 | 5.810545115 | 0.425268471 | 0.604966425 |
| IUGR_19 | IUGR | 4.77358074 | 5.611709162 | 0.837559118 | 0.69436346 |
| IUGR_20 | IUGR | 4.370157039 | 6.065499312 | 1.107791697 | 0.506970176 |
| IUGR_21 | IUGR | 6.672519015 | 7.851726853 | 0.224583626 | 0.146985449 |
| IUGR_22 | IUGR | 5.31937896 | 4.506187846 | 0.573738291 | 1.494107808 |
| IUGR_23 | IUGR | 5.554022564 | 6.549008152 | 0.487617262 | 0.362603296 |
| IUGR_24 | IUGR | 4.360201451 | 4.709827303 | 1.115462659 | 1.29741929 |
| IUGR_25 | IUGR | 5.06449382 | 5.622454507 | 0.684607902 | 0.689210979 |
| IUGR_26 | IUGR | 5.588738072 | 6.871160478 | 0.476023795 | 0.290037554 |
| IUGR_27 | IUGR | 4.579205138 | 6.051424015 | 0.958359291 | 0.511940512 |
| IUGR_28 | IUGR | 5.800558415 | 5.137804178 | 0.411021354 | 0.964376178 |
| IUGR_29 | IUGR | 5.019489048 | 5.427757033 | 0.706300793 | 0.788791037 |
| IUGR_30 | IUGR | 5.266645 | 6.543678199 | 0.595097792 | 0.363945391 |
| IUGR_31 | IUGR | 5.01863471 | 5.415706695 | 0.706719175 | 0.795407131 |
| IUGR_32 | IUGR | 6.981822548 | 6.173166872 | 0.181246008 | 0.470512588 |
| IUGR_33 | IUGR | 5.48920222 | 6.036457472 | 0.510025558 | 0.517279034 |
| IUGR_34 | IUGR | 4.653831257 | 5.478719722 | 0.910046654 | 0.761413669 |
| IUGR_35 | IUGR | 6.15803593 | 7.715155271 | 0.320813718 | 0.161579584 |
| IUGR_36 | IUGR | 4.52332508 | 6.507135218 | 0.996207785 | 0.373281749 |
| IUGR_37 | IUGR | 5.667090876 | 4.379885931 | 0.450860427 | 1.630807126 |
| IUGR_38 | IUGR | 3.932263901 | 6.149163452 | 1.500641924 | 0.478406417 |
| IUGR_39 | IUGR | 4.437451161 | 5.470570828 | 1.057305659 | 0.765726594 |
| IUGR_40 | IUGR | 5.657488989 | 6.681946668 | 0.453871146 | 0.330684064 |
| IUGR_41 | IUGR | 6.090773264 | 5.365983409 | 0.336125152 | 0.823299176 |
| IUGR_42 | IUGR | 5.637094625 | 5.908210847 | 0.460332767 | 0.565367654 |
| IUGR_43 | IUGR | 5.407481374 | 6.403989823 | 0.539749645 | 0.400946673 |
| IUGR_44 | IUGR | 5.259117044 | 6.692604155 | 0.598211117 | 0.328250233 |
| IUGR_45 | IUGR | 4.317182408 | 5.039762874 | 1.149224984 | 1.032190475 |
| IUGR_46 | IUGR | 4.924124633 | 5.732399011 | 0.754566019 | 0.638639231 |
| IUGR_47 | IUGR | 5.131488983 | 5.620043751 | 0.653543281 | 0.69036362 |
| IUGR_48 | IUGR | 6.345697781 | 5.477336614 | 0.28168329 | 0.762143984 |
| IUGR_49 | IUGR | 5.774894632 | 7.412363392 | 0.418398342 | 0.199313136 |
| IUGR_50 | IUGR | 4.089567876 | 6.323985369 | 1.345624421 | 0.423809176 |
| IUGR_51 | IUGR | 5.759267176 | 4.991292837 | 0.422955121 | 1.067457965 |
| IUGR_52 | IUGR | 5.191934176 | 6.734289558 | 0.626727192 | 0.318901445 |
| IUGR_53 | IUGR | 4.9584624 | 7.697724958 | 0.736818541 | 0.163543592 |
| IUGR_54 | IUGR | 5.989341031 | 6.825972208 | 0.360607833 | 0.299265917 |
| IUGR_55 | IUGR | 6.324799618 | 4.784504027 | 0.28579331 | 1.231970806 |
| IUGR_56 | IUGR | 6.245024095 | 5.612612742 | 0.302041685 | 0.693928707 |
| IUGR_57 | IUGR | 4.828625981 | 7.013528919 | 0.80620447 | 0.262782877 |
| IUGR_58 | IUGR | 5.252630099 | 5.433864428 | 0.600906974 | 0.785458888 |
| IUGR_59 | IUGR | 5.765010745 | 6.355055543 | 0.421274625 | 0.414779516 |
| IUGR_60 | IUGR | 6.280436102 | 6.619707243 | 0.294718099 | 0.345262333 |
| IUGR_61 | IUGR | 5.11666061 | 5.258455623 | 0.660295198 | 0.887006434 |
| IUGR_62 | IUGR | 5.351472819 | 5.952379715 | 0.561115959 | 0.548320904 |
| IUGR_63 | IUGR | 4.614932021 | 3.406986128 | 0.934917941 | 3.200918554 |
| IUGR_64 | IUGR | 4.543034701 | 5.180489887 | 0.982690469 | 0.936260702 |
| IUGR_65 | IUGR | 6.150020658 | 5.797945479 | 0.322601044 | 0.610272978 |
| IUGR_66 | IUGR | 6.584992023 | 5.001773454 | 0.238630714 | 1.059731398 |
| IUGR_67 | IUGR | 5.442391903 | 7.305929043 | 0.526845469 | 0.214573386 |
| IUGR_68 | IUGR | 6.302826318 | 4.855886898 | 0.290179469 | 1.172497786 |
| IUGR_69 | IUGR | 5.78930882 | 5.647964411 | 0.414238873 | 0.677131381 |
| IUGR_70 | IUGR | 4.983904196 | 6.104592462 | 0.723938714 | 0.493417105 |
| IUGR_71 | IUGR | 5.789116484 | 7.153018631 | 0.414294101 | 0.238564861 |
| IUGR_72 | IUGR | 6.730429253 | 4.851310279 | 0.215747301 | 1.176223172 |
| IUGR_73 | IUGR | 5.471339169 | 6.930531002 | 0.516379811 | 0.27834404 |
| IUGR_74 | IUGR | 6.751714925 | 6.008186449 | 0.21258751 | 0.527515594 |
| IUGR_75 | IUGR | 3.404203917 | 5.214793079 | 2.163908884 | 0.914261654 |
| IUGR_76 | IUGR | 6.157522004 | 6.369682779 | 0.320928021 | 0.410595385 |
| IUGR_77 | IUGR | 5.569637655 | 6.159247756 | 0.482367969 | 0.47507406 |
| IUGR_78 | IUGR | 5.26079412 | 5.519826498 | 0.597516124 | 0.740024838 |
| IUGR_79 | IUGR | 5.573408621 | 6.055841668 | 0.481108785 | 0.510375305 |
| IUGR_80 | IUGR | 3.909944868 | 5.691749123 | 1.524037922 | 0.656889666 |
| IUGR_81 | IUGR | 5.32426249 | 6.090813876 | 0.571799467 | 0.498152103 |
| IUGR_82 | IUGR | 5.785690057 | 6.52970454 | 0.415279227 | 0.367487621 |
| IUGR_83 | IUGR | 6.682315236 | 7.268813453 | 0.223063819 | 0.220165244 |
| IUGR_84 | IUGR | 5.085383825 | 5.009747601 | 0.674766308 | 1.053890148 |
| IUGR_85 | IUGR | 4.853205118 | 7.7064267 | 0.79258554 | 0.162560133 |
| IUGR_86 | IUGR | 5.098594365 | 4.43832976 | 0.668615778 | 1.566063093 |
| IUGR_87 | IUGR | 6.232321694 | 5.878571924 | 0.304712793 | 0.577102775 |
| IUGR_88 | IUGR | 5.763000888 | 6.470653765 | 0.421861923 | 0.382841288 |
| IUGR_89 | IUGR | 5.076191837 | 6.224793494 | 0.679079239 | 0.453973063 |
| IUGR_90 | IUGR | 5.910613946 | 5.501840384 | 0.380832821 | 0.749308495 |
| IUGR_91 | IUGR | 5.577662039 | 5.8335022 | 0.479692447 | 0.595416001 |
| IUGR_92 | IUGR | 6.274915992 | 5.605599252 | 0.295847924 | 0.697310371 |
| IUGR_93 | IUGR | 4.938357525 | 5.528508194 | 0.747158456 | 0.735584968 |
| IUGR_94 | IUGR | 5.237870283 | 6.679681678 | 0.607086243 | 0.331203637 |
| IUGR_95 | IUGR | 5.186313477 | 6.285612389 | 0.629173666 | 0.435232956 |
| IUGR_96 | IUGR | 4.329188041 | 5.445672324 | 1.139701193 | 0.779056449 |
| IUGR_97 | IUGR | 5.736896222 | 6.7196799 | 0.42956473 | 0.322147253 |
| IUGR_98 | IUGR | 5.708844218 | 6.245839617 | 0.437998991 | 0.447398548 |
| IUGR_99 | IUGR | 5.504090765 | 6.650289695 | 0.504789184 | 0.338020442 |
| IUGR_100 | IUGR | 5.312330293 | 6.503703074 | 0.576548299 | 0.374170837 |
| AGA_1 | AGA | 3.367703406 | 4.336803991 | 2.219354706 | 1.680240917 |
| AGA_2 | AGA | 4.163483742 | 4.551855168 | 1.278418267 | 1.44755376 |
| AGA_3 | AGA | 4.225828387 | 5.597834884 | 1.224349362 | 0.701073308 |
| AGA_4 | AGA | 3.858178185 | 5.488296212 | 1.579716295 | 0.756376206 |
| AGA_5 | AGA | 4.370971431 | 4.983278725 | 1.107166533 | 1.073404151 |
| AGA_6 | AGA | 4.823240685 | 5.093861907 | 0.809219496 | 0.994201503 |
| AGA_7 | AGA | 6.008948721 | 6.022131917 | 0.355739961 | 0.522441056 |
| AGA_8 | AGA | 4.63966225 | 4.526742889 | 0.919028444 | 1.472971185 |
| AGA_9 | AGA | 4.706040313 | 5.437677905 | 0.877702156 | 0.783385425 |
| AGA_10 | AGA | 4.440443267 | 4.838245878 | 1.055115111 | 1.186922896 |
| AGA_11 | AGA | 2.964983028 | 4.825855037 | 2.933983265 | 1.197160895 |
| AGA_12 | AGA | 4.4787889 | 5.879021482 | 1.027440444 | 0.576922972 |
| AGA_13 | AGA | 4.548184168 | 5.660333079 | 0.979189167 | 0.671350939 |
| AGA_14 | AGA | 6.47059369 | 5.650807709 | 0.258323353 | 0.675798188 |
| AGA_15 | AGA | 4.346111228 | 6.044383046 | 1.126410308 | 0.514445107 |
| AGA_16 | AGA | 4.741237874 | 5.016803073 | 0.856547878 | 1.048748701 |
| AGA_17 | AGA | 4.472230584 | 5.545562377 | 1.032121695 | 0.726940767 |
| AGA_18 | AGA | 3.56505757 | 4.751786595 | 1.935607051 | 1.26022864 |
| AGA_19 | AGA | 5.414258252 | 5.259333082 | 0.537220185 | 0.886467113 |
| AGA_20 | AGA | 5.101546426 | 4.895885557 | 0.667249046 | 1.140436833 |
| AGA_21 | AGA | 5.132825558 | 5.077596772 | 0.652938091 | 1.005473685 |
| AGA_22 | AGA | 3.772490036 | 5.47612562 | 1.676385135 | 0.762783994 |
| AGA_23 | AGA | 5.622235449 | 4.345423453 | 0.465098509 | 1.670232153 |
| AGA_24 | AGA | 3.37851915 | 6.673909821 | 2.202778634 | 0.332531353 |
| AGA_25 | AGA | 4.969485675 | 4.195186095 | 0.731210146 | 1.853543536 |
| AGA_26 | AGA | 6.252364501 | 4.02864911 | 0.300508805 | 2.080345267 |
| AGA_27 | AGA | 3.70757094 | 5.926488699 | 1.753542904 | 0.558250057 |
| AGA_28 | AGA | 4.046961816 | 5.633330155 | 1.385956381 | 0.684034948 |
| AGA_29 | AGA | 4.579721092 | 5.499295854 | 0.958016612 | 0.750631242 |
| AGA_30 | AGA | 4.097219477 | 5.502676407 | 1.338506545 | 0.748874406 |
| AGA_31 | AGA | 3.259469255 | 4.990202582 | 2.392260363 | 1.068264956 |
| AGA_32 | AGA | 4.55485038 | 4.282196503 | 0.974675098 | 1.745058733 |
| AGA_33 | AGA | 3.650157029 | 5.060643647 | 1.824734594 | 1.017358711 |
| AGA_34 | AGA | 4.878873945 | 4.458270631 | 0.778608352 | 1.544565943 |
| AGA_35 | AGA | 3.764460613 | 5.780095787 | 1.685741189 | 0.617870462 |
| AGA_36 | AGA | 5.739947524 | 4.882354095 | 0.42865716 | 1.151183646 |
| AGA_37 | AGA | 3.873397366 | 4.339602243 | 1.563139249 | 1.676985081 |
| AGA_38 | AGA | 4.242350787 | 4.742891327 | 1.210407542 | 1.268022873 |
| AGA_39 | AGA | 5.150813774 | 5.330345163 | 0.644847488 | 0.843890041 |
| AGA_40 | AGA | 3.515308547 | 4.549020358 | 2.003517516 | 1.450400913 |
| AGA_41 | AGA | 4.681967948 | 4.342223684 | 0.892470088 | 1.673940691 |
| AGA_42 | AGA | 5.545714203 | 5.194949769 | 0.490433511 | 0.926923594 |
| AGA_43 | AGA | 3.214013412 | 5.195973257 | 2.468834722 | 0.926266242 |
| AGA_44 | AGA | 4.647707087 | 4.59444546 | 0.913917969 | 1.405444649 |
| AGA_45 | AGA | 4.707906235 | 4.623169356 | 0.876567706 | 1.377739126 |
| AGA_46 | AGA | 5.125458297 | 5.18563995 | 0.656280909 | 0.932924442 |
| AGA_47 | AGA | 3.510439431 | 3.841532527 | 2.010290839 | 2.368444014 |
| AGA_48 | AGA | 3.44363471 | 3.87402898 | 2.105567258 | 2.315691577 |
| AGA_49 | AGA | 4.917553252 | 4.425244623 | 0.758010857 | 1.580331779 |
| AGA_50 | AGA | 4.737587739 | 4.829242279 | 0.858717758 | 1.19435343 |
| AGA_51 | AGA | 4.70039428 | 5.248726052 | 0.881143801 | 0.893008643 |
| AGA_52 | AGA | 4.777158568 | 6.180284974 | 0.835484577 | 0.468196847 |
| AGA_53 | AGA | 3.955980223 | 5.686127699 | 1.476174677 | 0.659454212 |
| AGA_54 | AGA | 4.685802958 | 4.872049176 | 0.890100851 | 1.159435786 |
| AGA_55 | AGA | 4.734457979 | 4.984787034 | 0.860582669 | 1.072282514 |
| AGA_56 | AGA | 3.928518866 | 4.197976508 | 1.504542441 | 1.849961938 |
| AGA_57 | AGA | 5.992619609 | 4.985189491 | 0.359789268 | 1.07198343 |
| AGA_58 | AGA | 4.879066337 | 4.769073089 | 0.778504527 | 1.245218579 |
| AGA_59 | AGA | 3.546957202 | 5.258174848 | 1.960044579 | 0.887179078 |
| AGA_60 | AGA | 5.025242887 | 4.338215245 | 0.703489494 | 1.678598098 |
| AGA_61 | AGA | 3.720254664 | 5.415477211 | 1.738193874 | 0.795533663 |
| AGA_62 | AGA | 5.129667683 | 6.22619113 | 0.654368854 | 0.453533482 |
| AGA_63 | AGA | 5.426876463 | 4.912991881 | 0.532541996 | 1.126994297 |
| AGA_64 | AGA | 3.843454145 | 5.321369378 | 1.595921317 | 0.849156704 |
| AGA_65 | AGA | 5.270700903 | 5.552115193 | 0.59342712 | 0.723646441 |
| AGA_66 | AGA | 4.830224742 | 4.679023622 | 0.805311549 | 1.325418971 |
| AGA_67 | AGA | 5.157648128 | 5.179273985 | 0.641799932 | 0.937050114 |
| AGA_68 | AGA | 6.017434386 | 5.010073921 | 0.353653706 | 1.053651798 |
| AGA_69 | AGA | 4.303689507 | 5.078140879 | 1.160023605 | 1.005094546 |
| AGA_70 | AGA | 3.897011069 | 4.381592173 | 1.537762391 | 1.628879548 |
| AGA_71 | AGA | 3.788388456 | 5.019608139 | 1.658012879 | 1.046711575 |
| AGA_72 | AGA | 3.847351772 | 5.398398633 | 1.591615548 | 0.805007128 |
| AGA_73 | AGA | 4.438318632 | 6.160914886 | 1.056670107 | 0.474525398 |
| AGA_74 | AGA | 4.77292158 | 5.767416661 | 0.837941882 | 0.623324548 |
| AGA_75 | AGA | 4.721352639 | 6.722545966 | 0.868435755 | 0.321507908 |
| AGA_76 | AGA | 5.161746599 | 4.38612195 | 0.639979266 | 1.623773209 |
| AGA_77 | AGA | 4.510401514 | 5.697856509 | 1.005171838 | 0.654114721 |
| AGA_78 | AGA | 5.662827262 | 5.146673605 | 0.452194832 | 0.958465556 |
| AGA_79 | AGA | 4.288274533 | 6.751842347 | 1.172484732 | 0.315044985 |
| AGA_80 | AGA | 6.676135333 | 4.353361372 | 0.224021381 | 1.661067528 |
| AGA_81 | AGA | 5.000533878 | 4.328222526 | 0.715641914 | 1.690265141 |
| AGA_82 | AGA | 3.814273955 | 4.520485884 | 1.628529343 | 1.479373372 |
| AGA_83 | AGA | 3.643286002 | 3.300883421 | 1.833445864 | 3.445202244 |
| AGA_84 | AGA | 4.885977932 | 4.579395983 | 0.774783823 | 1.420182283 |
| AGA_85 | AGA | 4.321229772 | 4.392693871 | 1.146005444 | 1.616393243 |
| AGA_86 | AGA | 5.071200395 | 5.120315029 | 0.681432789 | 0.976138028 |
| AGA_87 | AGA | 4.8785901 | 5.273404781 | 0.778761555 | 0.877862758 |
| AGA_88 | AGA | 4.44173687 | 6.500936671 | 1.054169458 | 0.374889007 |
| AGA_89 | AGA | 3.822565026 | 5.760339071 | 1.619197137 | 0.626389974 |
| AGA_90 | AGA | 3.28812222 | 4.538477075 | 2.345217048 | 1.461039336 |
| AGA_91 | AGA | 4.142788038 | 4.281268263 | 1.296889563 | 1.746181877 |
| AGA_92 | AGA | 5.185119035 | 5.393535337 | 0.629694789 | 0.80772537 |
| AGA_93 | AGA | 4.671274995 | 3.943813434 | 0.899109463 | 2.206345403 |
| AGA_94 | AGA | 3.503408977 | 6.465167013 | 2.020111175 | 0.384300054 |
| AGA_95 | AGA | 4.638544741 | 5.943552097 | 0.919740598 | 0.551686277 |
| AGA_96 | AGA | 4.808253904 | 4.624659478 | 0.817669519 | 1.37631683 |
| AGA_97 | AGA | 3.792914051 | 3.629492377 | 1.652820002 | 2.743428012 |
| AGA_98 | AGA | 4.622980085 | 6.083097899 | 0.929717028 | 0.500823511 |
| AGA_99 | AGA | 4.546566975 | 4.908368124 | 0.980287407 | 1.130612046 |
| AGA_100 | AGA | 3.585623762 | 5.99025305 | 1.908209945 | 0.534113793 |
