# Supplementary material for: Adiponectin and leptin levels in mothers, fetuses, and neonates with intrauterine growth restriction compared to those with appropriate gestational age
Source: Front Endocrinol (Lausanne). 2026 May 26;17:1827618. doi: 10.3389/fendo.2026.1827618 (PMC13246382; doi:10.3389/fendo.2026.1827618)
Supplement: Supplementary file 2 [file Table2.docx]

| **Patient_ID**  **Supplementary Table 2:** Integrated qRT-PCR Expression (ΔCt and Relative Expression) and DNA Methylation Profiles of *ADIPOQ* and *LEP* Genes in IUGR Placental Samples | **Group** | **DeltaCt_ADIPOQ** | **DeltaCt_LEP** | **Relative_Expression_ADIPOQ** | **Relative_Expression_LEP** | **Methylation_ADIPOQ** | **Methylation_LEP** |
| --- | --- | --- | --- | --- | --- | --- | --- |
| IUGR_1 | IUGR | 5.897371322 | 6.286229888 | 0.384344612 | 0.435046708 | 52.24457873 | 76.05590893 |
| IUGR_2 | IUGR | 5.389388559 | 6.448627621 | 0.54656126 | 0.38873111 | 60.20499982 | 62.62267741 |
| IUGR_3 | IUGR | 6.01815083 | 6.866440995 | 0.353478124 | 0.290987907 | 65.0419496 | 76.95684736 |
| IUGR_4 | IUGR | 6.718423885 | 6.843041642 | 0.21755013 | 0.295745978 | 65.37584475 | 80.84510287 |
| IUGR_5 | IUGR | 5.3126773 | 4.897864506 | 0.57640964 | 1.138873565 | 61.39947623 | 73.30747923 |
| IUGR_6 | IUGR | 5.312690434 | 5.249739968 | 0.576404393 | 0.892381264 | 69.98279946 | 85.0143665 |
| IUGR_7 | IUGR | 6.763370252 | 6.412028214 | 0.210876965 | 0.398718896 | 56.45903656 | 63.80968641 |
| IUGR_8 | IUGR | 6.113947783 | 6.411028761 | 0.330768989 | 0.398995211 | 63.86096412 | 60.04276237 |
| IUGR_9 | IUGR | 5.124420491 | 6.412038149 | 0.656753177 | 0.39871615 | 65.96236505 | 55.77023801 |
| IUGR_10 | IUGR | 5.934048035 | 9.082185193 | 0.374696819 | 0.062642557 | 69.11551067 | 81.96835449 |
| IUGR_11 | IUGR | 5.129265846 | 6.456712409 | 0.654551142 | 0.386558774 | 70.69291902 | 75.23492525 |
| IUGR_12 | IUGR | 5.127416197 | 6.908452512 | 0.655390866 | 0.282636479 | 56.00286327 | 69.55532263 |
| IUGR_13 | IUGR | 5.693569817 | 6.763201411 | 0.4426609 | 0.312574217 | 52.72708663 | 72.23974901 |
| IUGR_14 | IUGR | 3.969375804 | 6.521113001 | 1.46253167 | 0.369682613 | 75.22141458 | 60.99608762 |
| IUGR_15 | IUGR | 4.120065734 | 5.747784604 | 1.317477146 | 0.631864664 | 67.6585121 | 89.56601584 |
| IUGR_16 | IUGR | 5.050169977 | 6.607175376 | 0.691438908 | 0.348274493 | 59.01210771 | 71.03376946 |
| IUGR_17 | IUGR | 4.689735104 | 5.381739828 | 0.887678134 | 0.814356423 | 77.4092158 | 70.87515836 |
| IUGR_18 | IUGR | 5.751397866 | 5.810545115 | 0.425268471 | 0.604966425 | 65.92539707 | 75.80613299 |
| IUGR_19 | IUGR | 4.77358074 | 5.611709162 | 0.837559118 | 0.69436346 | 74.43437747 | 73.84807385 |
| IUGR_20 | IUGR | 4.370157039 | 6.065499312 | 1.107791697 | 0.506970176 | 65.54014785 | 71.79107219 |
| IUGR_21 | IUGR | 6.672519015 | 7.851726853 | 0.224583626 | 0.146985449 | 81.4859834 | 63.67620436 |
| IUGR_22 | IUGR | 5.31937896 | 4.506187846 | 0.573738291 | 1.494107808 | 79.04272674 | 73.77174686 |
| IUGR_23 | IUGR | 5.554022564 | 6.549008152 | 0.487617262 | 0.362603296 | 63.00828681 | 85.05619597 |
| IUGR_24 | IUGR | 4.360201451 | 4.709827303 | 1.115462659 | 1.29741929 | 72.77256761 | 80.76336037 |
| IUGR_25 | IUGR | 5.06449382 | 5.622454507 | 0.684607902 | 0.689210979 | 70.1630076 | 82.74549301 |
| IUGR_26 | IUGR | 5.588738072 | 6.871160478 | 0.476023795 | 0.290037554 | 75.94905246 | 65.91027459 |
| IUGR_27 | IUGR | 4.579205138 | 6.051424015 | 0.958359291 | 0.511940512 | 57.28061232 | 62.08316144 |
| IUGR_28 | IUGR | 5.800558415 | 5.137804178 | 0.411021354 | 0.964376178 | 70.48841168 | 68.99370464 |
| IUGR_29 | IUGR | 5.019489048 | 5.427757033 | 0.706300793 | 0.788791037 | 73.46739589 | 70.4457993 |
| IUGR_30 | IUGR | 5.266645 | 6.543678199 | 0.595097792 | 0.363945391 | 50.93008411 | 78.75353215 |
| IUGR_31 | IUGR | 5.01863471 | 5.415706695 | 0.706719175 | 0.795407131 | 55.5339319 | 56.46028296 |
| IUGR_32 | IUGR | 6.981822548 | 6.173166872 | 0.181246008 | 0.470512588 | 48.68614258 | 82.23640256 |
| IUGR_33 | IUGR | 5.48920222 | 6.036457472 | 0.510025558 | 0.517279034 | 62.84474532 | 68.73593681 |
| IUGR_34 | IUGR | 4.653831257 | 5.478719722 | 0.910046654 | 0.761413669 | 70.74033805 | 66.58495144 |
| IUGR_35 | IUGR | 6.15803593 | 7.715155271 | 0.320813718 | 0.161579584 | 77.01885642 | 61.903165 |
| IUGR_36 | IUGR | 4.52332508 | 6.507135218 | 0.996207785 | 0.373281749 | 65.59275824 | 56.76114663 |
| IUGR_37 | IUGR | 5.667090876 | 4.379885931 | 0.450860427 | 1.630807126 | 78.02892436 | 76.58536467 |
| IUGR_38 | IUGR | 3.932263901 | 6.149163452 | 1.500641924 | 0.478406417 | 53.95918833 | 70.58654374 |
| IUGR_39 | IUGR | 4.437451161 | 5.470570828 | 1.057305659 | 0.765726594 | 51.37294049 | 59.6803128 |
| IUGR_40 | IUGR | 5.657488989 | 6.681946668 | 0.453871146 | 0.330684064 | 64.55561841 | 59.63936982 |
| IUGR_41 | IUGR | 6.090773264 | 5.365983409 | 0.336125152 | 0.823299176 | 68.07252359 | 67.31372241 |
| IUGR_42 | IUGR | 5.637094625 | 5.908210847 | 0.460332767 | 0.565367654 | 64.73844202 | 83.3521722 |
| IUGR_43 | IUGR | 5.407481374 | 6.403989823 | 0.539749645 | 0.400946673 | 48.4604632 | 67.92326919 |
| IUGR_44 | IUGR | 5.259117044 | 6.692604155 | 0.598211117 | 0.328250233 | 64.28703968 | 57.97485638 |
| IUGR_45 | IUGR | 4.317182408 | 5.039762874 | 1.149224984 | 1.032190475 | 54.564244 | 68.03405549 |
| IUGR_46 | IUGR | 4.924124633 | 5.732399011 | 0.754566019 | 0.638639231 | 70.35738039 | 67.81821144 |
| IUGR_47 | IUGR | 5.131488983 | 5.620043751 | 0.653543281 | 0.69036362 | 67.93278597 | 48.42490686 |
| IUGR_48 | IUGR | 6.345697781 | 5.477336614 | 0.28168329 | 0.762143984 | 57.48096171 | 69.56564107 |
| IUGR_49 | IUGR | 5.774894632 | 7.412363392 | 0.418398342 | 0.199313136 | 60.88906466 | 68.15252376 |
| IUGR_50 | IUGR | 4.089567876 | 6.323985369 | 1.345624421 | 0.423809176 | 56.52629182 | 75.56965092 |
| IUGR_51 | IUGR | 5.759267176 | 4.991292837 | 0.422955121 | 1.067457965 | 64.49856722 | 84.79164876 |
| IUGR_52 | IUGR | 5.191934176 | 6.734289558 | 0.626727192 | 0.318901445 | 72.64113856 | 79.01252024 |
| IUGR_53 | IUGR | 4.9584624 | 7.697724958 | 0.736818541 | 0.163543592 | 57.11419163 | 67.84889048 |
| IUGR_54 | IUGR | 5.989341031 | 6.825972208 | 0.360607833 | 0.299265917 | 69.03237212 | 61.14779273 |
| IUGR_55 | IUGR | 6.324799618 | 4.784504027 | 0.28579331 | 1.231970806 | 60.75793905 | 90.58687843 |
| IUGR_56 | IUGR | 6.245024095 | 5.612612742 | 0.302041685 | 0.693928707 | 58.65701734 | 70.47374747 |
| IUGR_57 | IUGR | 4.828625981 | 7.013528919 | 0.80620447 | 0.262782877 | 64.14375712 | 70.11143434 |
| IUGR_58 | IUGR | 5.252630099 | 5.433864428 | 0.600906974 | 0.785458888 | 56.71806142 | 69.8069993 |
| IUGR_59 | IUGR | 5.765010745 | 6.355055543 | 0.421274625 | 0.414779516 | 60.57080556 | 71.58467809 |
| IUGR_60 | IUGR | 6.280436102 | 6.619707243 | 0.294718099 | 0.345262333 | 55.41697686 | 68.8451167 |
| IUGR_61 | IUGR | 5.11666061 | 5.258455623 | 0.660295198 | 0.887006434 | 80.71780106 | 65.41070394 |
| IUGR_62 | IUGR | 5.351472819 | 5.952379715 | 0.561115959 | 0.548320904 | 65.28210842 | 65.62512847 |
| IUGR_63 | IUGR | 4.614932021 | 3.406986128 | 0.934917941 | 3.200918554 | 59.40219594 | 69.73797384 |
| IUGR_64 | IUGR | 4.543034701 | 5.180489887 | 0.982690469 | 0.936260702 | 66.71183929 | 65.65260183 |
| IUGR_65 | IUGR | 6.150020658 | 5.797945479 | 0.322601044 | 0.610272978 | 64.1013756 | 64.29723374 |
| IUGR_66 | IUGR | 6.584992023 | 5.001773454 | 0.238630714 | 1.059731398 | 63.2322432 | 70.85144182 |
| IUGR_67 | IUGR | 5.442391903 | 7.305929043 | 0.526845469 | 0.214573386 | 69.9133336 | 67.96018226 |
| IUGR_68 | IUGR | 6.302826318 | 4.855886898 | 0.290179469 | 1.172497786 | 71.06006168 | 82.03194391 |
| IUGR_69 | IUGR | 5.78930882 | 5.647964411 | 0.414238873 | 0.677131381 | 60.75599082 | 48.79224153 |
| IUGR_70 | IUGR | 4.983904196 | 6.104592462 | 0.723938714 | 0.493417105 | 60.39345407 | 78.73205482 |
| IUGR_71 | IUGR | 5.789116484 | 7.153018631 | 0.414294101 | 0.238564861 | 62.79958642 | 79.96868154 |
| IUGR_72 | IUGR | 6.730429253 | 4.851310279 | 0.215747301 | 1.176223172 | 46.58463068 | 53.41287814 |
| IUGR_73 | IUGR | 5.471339169 | 6.930531002 | 0.516379811 | 0.27834404 | 52.8784715 | 67.25849925 |
| IUGR_74 | IUGR | 6.751714925 | 6.008186449 | 0.21258751 | 0.527515594 | 75.93499414 | 67.02847307 |
| IUGR_75 | IUGR | 3.404203917 | 5.214793079 | 2.163908884 | 0.914261654 | 78.15974171 | 58.73990644 |
| IUGR_76 | IUGR | 6.157522004 | 6.369682779 | 0.320928021 | 0.410595385 | 63.00771168 | 63.7774665 |
| IUGR_77 | IUGR | 5.569637655 | 6.159247756 | 0.482367969 | 0.47507406 | 69.6124557 | 61.11539324 |
| IUGR_78 | IUGR | 5.26079412 | 5.519826498 | 0.597516124 | 0.740024838 | 67.49000124 | 84.01816355 |
| IUGR_79 | IUGR | 5.573408621 | 6.055841668 | 0.481108785 | 0.510375305 | 89.63104647 | 77.48542715 |
| IUGR_80 | IUGR | 3.909944868 | 5.691749123 | 1.524037922 | 0.656889666 | 73.95659929 | 80.17244076 |
| IUGR_81 | IUGR | 5.32426249 | 6.090813876 | 0.571799467 | 0.498152103 | 63.97665927 | 75.77337651 |
| IUGR_82 | IUGR | 5.785690057 | 6.52970454 | 0.415279227 | 0.367487621 | 57.35567648 | 60.96758583 |
| IUGR_83 | IUGR | 6.682315236 | 7.268813453 | 0.223063819 | 0.220165244 | 52.14842944 | 65.80383787 |
| IUGR_84 | IUGR | 5.085383825 | 5.009747601 | 0.674766308 | 1.053890148 | 66.62770909 | 73.91499649 |
| IUGR_85 | IUGR | 4.853205118 | 7.7064267 | 0.79258554 | 0.162560133 | 58.94919404 | 60.22297753 |
| IUGR_86 | IUGR | 5.098594365 | 4.43832976 | 0.668615778 | 1.566063093 | 53.62197032 | 75.70398744 |
| IUGR_87 | IUGR | 6.232321694 | 5.878571924 | 0.304712793 | 0.577102775 | 59.82741693 | 68.07739681 |
| IUGR_88 | IUGR | 5.763000888 | 6.470653765 | 0.421861923 | 0.382841288 | 56.34761597 | 67.00143354 |
| IUGR_89 | IUGR | 5.076191837 | 6.224793494 | 0.679079239 | 0.453973063 | 78.49713308 | 75.68767975 |
| IUGR_90 | IUGR | 5.910613946 | 5.501840384 | 0.380832821 | 0.749308495 | 72.05311806 | 73.55410649 |
| IUGR_91 | IUGR | 5.577662039 | 5.8335022 | 0.479692447 | 0.595416001 | 64.93621887 | 67.11227067 |
| IUGR_92 | IUGR | 6.274915992 | 5.605599252 | 0.295847924 | 0.697310371 | 76.83955311 | 79.27463843 |
| IUGR_93 | IUGR | 4.938357525 | 5.528508194 | 0.747158456 | 0.735584968 | 65.61894646 | 61.35149338 |
| IUGR_94 | IUGR | 5.237870283 | 6.679681678 | 0.607086243 | 0.331203637 | 58.10972639 | 74.92748486 |
| IUGR_95 | IUGR | 5.186313477 | 6.285612389 | 0.629173666 | 0.435232956 | 77.18499262 | 74.74481006 |
| IUGR_96 | IUGR | 4.329188041 | 5.445672324 | 1.139701193 | 0.779056449 | 69.31128035 | 67.52362849 |
| IUGR_97 | IUGR | 5.736896222 | 6.7196799 | 0.42956473 | 0.322147253 | 56.70203077 | 72.60906418 |
| IUGR_98 | IUGR | 5.708844218 | 6.245839617 | 0.437998991 | 0.447398548 | 63.47729058 | 59.99109139 |
| IUGR_99 | IUGR | 5.504090765 | 6.650289695 | 0.504789184 | 0.338020442 | 57.99505397 | 77.39221615 |
| IUGR_100 | IUGR | 5.312330293 | 6.503703074 | 0.576548299 | 0.374170837 | 53.93760215 | 68.52078291 |
| AGA_1 | AGA | 3.367703406 | 4.336803991 | 2.219354706 | 1.680240917 | 52.40942038 | 45.81821584 |
| AGA_2 | AGA | 4.163483742 | 4.551855168 | 1.278418267 | 1.44755376 | 60.27533312 | 58.39207381 |
| AGA_3 | AGA | 4.225828387 | 5.597834884 | 1.224349362 | 0.701073308 | 33.81145941 | 44.36525048 |
| AGA_4 | AGA | 3.858178185 | 5.488296212 | 1.579716295 | 0.756376206 | 49.50375389 | 38.73230963 |
| AGA_5 | AGA | 4.370971431 | 4.983278725 | 1.107166533 | 1.073404151 | 39.79485945 | 37.54696661 |
| AGA_6 | AGA | 4.823240685 | 5.093861907 | 0.809219496 | 0.994201503 | 41.10299693 | 54.84807961 |
| AGA_7 | AGA | 6.008948721 | 6.022131917 | 0.355739961 | 0.522441056 | 40.26084861 | 39.75656518 |
| AGA_8 | AGA | 4.63966225 | 4.526742889 | 0.919028444 | 1.472971185 | 38.08807384 | 64.03835346 |
| AGA_9 | AGA | 4.706040313 | 5.437677905 | 0.877702156 | 0.783385425 | 45.38817302 | 33.34456474 |
| AGA_10 | AGA | 4.440443267 | 4.838245878 | 1.055115111 | 1.186922896 | 38.35239907 | 63.57165095 |
| AGA_11 | AGA | 2.964983028 | 4.825855037 | 2.933983265 | 1.197160895 | 47.16365461 | 51.68813974 |
| AGA_12 | AGA | 4.4787889 | 5.879021482 | 1.027440444 | 0.576922972 | 44.59809512 | 49.22629511 |
| AGA_13 | AGA | 4.548184168 | 5.660333079 | 0.979189167 | 0.671350939 | 43.08841563 | 45.64064731 |
| AGA_14 | AGA | 6.47059369 | 5.650807709 | 0.258323353 | 0.675798188 | 37.7394907 | 53.19308891 |
| AGA_15 | AGA | 4.346111228 | 6.044383046 | 1.126410308 | 0.514445107 | 40.38582936 | 49.69892238 |
| AGA_16 | AGA | 4.741237874 | 5.016803073 | 0.856547878 | 1.048748701 | 51.04312981 | 58.82641506 |
| AGA_17 | AGA | 4.472230584 | 5.545562377 | 1.032121695 | 0.726940767 | 49.0073375 | 50.91382119 |
| AGA_18 | AGA | 3.56505757 | 4.751786595 | 1.935607051 | 1.26022864 | 37.17955804 | 51.20241409 |
| AGA_19 | AGA | 5.414258252 | 5.259333082 | 0.537220185 | 0.886467113 | 45.79465844 | 47.0911023 |
| AGA_20 | AGA | 5.101546426 | 4.895885557 | 0.667249046 | 1.140436833 | 51.01109699 | 49.54443501 |
| AGA_21 | AGA | 5.132825558 | 5.077596772 | 0.652938091 | 1.005473685 | 31.64475775 | 52.46241415 |
| AGA_22 | AGA | 3.772490036 | 5.47612562 | 1.676385135 | 0.762783994 | 49.34688154 | 36.31865286 |
| AGA_23 | AGA | 5.622235449 | 4.345423453 | 0.465098509 | 1.670232153 | 39.69900993 | 39.21451662 |
| AGA_24 | AGA | 3.37851915 | 6.673909821 | 2.202778634 | 0.332531353 | 49.56478935 | 55.94611275 |
| AGA_25 | AGA | 4.969485675 | 4.195186095 | 0.731210146 | 1.853543536 | 38.89392675 | 51.36692351 |
| AGA_26 | AGA | 6.252364501 | 4.02864911 | 0.300508805 | 2.080345267 | 30.56094319 | 48.52813331 |
| AGA_27 | AGA | 3.70757094 | 5.926488699 | 1.753542904 | 0.558250057 | 31.9796605 | 50.14747146 |
| AGA_28 | AGA | 4.046961816 | 5.633330155 | 1.385956381 | 0.684034948 | 45.38467957 | 52.78065364 |
| AGA_29 | AGA | 4.579721092 | 5.499295854 | 0.958016612 | 0.750631242 | 47.07778001 | 45.68192256 |
| AGA_30 | AGA | 4.097219477 | 5.502676407 | 1.338506545 | 0.748874406 | 37.765467 | 43.7735622 |
| AGA_31 | AGA | 3.259469255 | 4.990202582 | 2.392260363 | 1.068264956 | 50.10873967 | 51.56676204 |
| AGA_32 | AGA | 4.55485038 | 4.282196503 | 0.974675098 | 1.745058733 | 31.7078395 | 42.17301778 |
| AGA_33 | AGA | 3.650157029 | 5.060643647 | 1.824734594 | 1.017358711 | 44.47136161 | 53.26602205 |
| AGA_34 | AGA | 4.878873945 | 4.458270631 | 0.778608352 | 1.544565943 | 35.3118704 | 36.37933117 |
| AGA_35 | AGA | 3.764460613 | 5.780095787 | 1.685741189 | 0.617870462 | 39.78531114 | 58.2332451 |
| AGA_36 | AGA | 5.739947524 | 4.882354095 | 0.42865716 | 1.151183646 | 45.37918937 | 53.78077986 |
| AGA_37 | AGA | 3.873397366 | 4.339602243 | 1.563139249 | 1.676985081 | 38.11669308 | 52.04823787 |
| AGA_38 | AGA | 4.242350787 | 4.742891327 | 1.210407542 | 1.268022873 | 41.92355565 | 57.86152787 |
| AGA_39 | AGA | 5.150813774 | 5.330345163 | 0.644847488 | 0.843890041 | 53.05034247 | 63.32379556 |
| AGA_40 | AGA | 3.515308547 | 4.549020358 | 2.003517516 | 1.450400913 | 40.38486504 | 58.11496052 |
| AGA_41 | AGA | 4.681967948 | 4.342223684 | 0.892470088 | 1.673940691 | 51.6855369 | 35.27300615 |
| AGA_42 | AGA | 5.545714203 | 5.194949769 | 0.490433511 | 0.926923594 | 35.96234516 | 39.76338427 |
| AGA_43 | AGA | 3.214013412 | 5.195973257 | 2.468834722 | 0.926266242 | 49.23843342 | 45.00145138 |
| AGA_44 | AGA | 4.647707087 | 4.59444546 | 0.913917969 | 1.405444649 | 56.53254897 | 50.2087284 |
| AGA_45 | AGA | 4.707906235 | 4.623169356 | 0.876567706 | 1.377739126 | 25.226844 | 54.14127216 |
| AGA_46 | AGA | 5.125458297 | 5.18563995 | 0.656280909 | 0.932924442 | 38.62483796 | 44.19404949 |
| AGA_47 | AGA | 3.510439431 | 3.841532527 | 2.010290839 | 2.368444014 | 49.61657702 | 51.49413412 |
| AGA_48 | AGA | 3.44363471 | 3.87402898 | 2.105567258 | 2.315691577 | 43.37563691 | 43.95693654 |
| AGA_49 | AGA | 4.917553252 | 4.425244623 | 0.758010857 | 1.580331779 | 47.96916699 | 45.10785758 |
| AGA_50 | AGA | 4.737587739 | 4.829242279 | 0.858717758 | 1.19435343 | 40.16811851 | 38.74671123 |
| AGA_51 | AGA | 4.70039428 | 5.248726052 | 0.881143801 | 0.893008643 | 45.6927183 | 42.61413403 |
| AGA_52 | AGA | 4.777158568 | 6.180284974 | 0.835484577 | 0.468196847 | 43.75458212 | 39.18652316 |
| AGA_53 | AGA | 3.955980223 | 5.686127699 | 1.476174677 | 0.659454212 | 54.34225649 | 42.19301398 |
| AGA_54 | AGA | 4.685802958 | 4.872049176 | 0.890100851 | 1.159435786 | 47.03536675 | 58.42913437 |
| AGA_55 | AGA | 4.734457979 | 4.984787034 | 0.860582669 | 1.072282514 | 47.7008213 | 42.40480889 |
| AGA_56 | AGA | 3.928518866 | 4.197976508 | 1.504542441 | 1.849961938 | 41.70498427 | 71.05905652 |
| AGA_57 | AGA | 5.992619609 | 4.985189491 | 0.359789268 | 1.07198343 | 41.09915021 | 53.94654321 |
| AGA_58 | AGA | 4.879066337 | 4.769073089 | 0.778504527 | 1.245218579 | 41.5395345 | 51.47868899 |
| AGA_59 | AGA | 3.546957202 | 5.258174848 | 1.960044579 | 0.887179078 | 48.15561714 | 43.13313776 |
| AGA_60 | AGA | 5.025242887 | 4.338215245 | 0.703489494 | 1.678598098 | 41.63212415 | 55.60247904 |
| AGA_61 | AGA | 3.720254664 | 5.415477211 | 1.738193874 | 0.795533663 | 47.31819886 | 45.39489739 |
| AGA_62 | AGA | 5.129667683 | 6.22619113 | 0.654368854 | 0.453533482 | 61.60320639 | 50.97607852 |
| AGA_63 | AGA | 5.426876463 | 4.912991881 | 0.532541996 | 1.126994297 | 51.96899763 | 70.48067631 |
| AGA_64 | AGA | 3.843454145 | 5.321369378 | 1.595921317 | 0.849156704 | 42.39181174 | 49.2315208 |
| AGA_65 | AGA | 5.270700903 | 5.552115193 | 0.59342712 | 0.723646441 | 54.60971138 | 59.19418661 |
| AGA_66 | AGA | 4.830224742 | 4.679023622 | 0.805311549 | 1.325418971 | 41.73539702 | 44.3745886 |
| AGA_67 | AGA | 5.157648128 | 5.179273985 | 0.641799932 | 0.937050114 | 28.69500372 | 49.72009208 |
| AGA_68 | AGA | 6.017434386 | 5.010073921 | 0.353653706 | 1.053651798 | 36.93530951 | 64.16640509 |
| AGA_69 | AGA | 4.303689507 | 5.078140879 | 1.160023605 | 1.005094546 | 30.03366463 | 44.98426354 |
| AGA_70 | AGA | 3.897011069 | 4.381592173 | 1.537762391 | 1.628879548 | 42.18789213 | 64.49958846 |
| AGA_71 | AGA | 3.788388456 | 5.019608139 | 1.658012879 | 1.046711575 | 45.14734703 | 55.66201548 |
| AGA_72 | AGA | 3.847351772 | 5.398398633 | 1.591615548 | 0.805007128 | 58.4114985 | 45.50026579 |
| AGA_73 | AGA | 4.438318632 | 6.160914886 | 1.056670107 | 0.474525398 | 47.61541899 | 55.05926191 |
| AGA_74 | AGA | 4.77292158 | 5.767416661 | 0.837941882 | 0.623324548 | 43.24719577 | 57.7804356 |
| AGA_75 | AGA | 4.721352639 | 6.722545966 | 0.868435755 | 0.321507908 | 51.63524465 | 54.9744797 |
| AGA_76 | AGA | 5.161746599 | 4.38612195 | 0.639979266 | 1.623773209 | 27.31091753 | 37.43820224 |
| AGA_77 | AGA | 4.510401514 | 5.697856509 | 1.005171838 | 0.654114721 | 46.88491646 | 44.18290259 |
| AGA_78 | AGA | 5.662827262 | 5.146673605 | 0.452194832 | 0.958465556 | 51.16692155 | 48.01985092 |
| AGA_79 | AGA | 4.288274533 | 6.751842347 | 1.172484732 | 0.315044985 | 33.17131003 | 49.40453257 |
| AGA_80 | AGA | 6.676135333 | 4.353361372 | 0.224021381 | 1.661067528 | 54.15003235 | 54.96537678 |
| AGA_81 | AGA | 5.000533878 | 4.328222526 | 0.715641914 | 1.690265141 | 47.70797126 | 51.42160801 |
| AGA_82 | AGA | 3.814273955 | 4.520485884 | 1.628529343 | 1.479373372 | 41.67769669 | 39.31724513 |
| AGA_83 | AGA | 3.643286002 | 3.300883421 | 1.833445864 | 3.445202244 | 50.06225493 | 53.04158281 |
| AGA_84 | AGA | 4.885977932 | 4.579395983 | 0.774783823 | 1.420182283 | 63.16554286 | 54.88468596 |
| AGA_85 | AGA | 4.321229772 | 4.392693871 | 1.146005444 | 1.616393243 | 46.45493004 | 54.47832358 |
| AGA_86 | AGA | 5.071200395 | 5.120315029 | 0.681432789 | 0.976138028 | 46.98576469 | 58.6462458 |
| AGA_87 | AGA | 4.8785901 | 5.273404781 | 0.778761555 | 0.877862758 | 41.3251128 | 56.67137724 |
| AGA_88 | AGA | 4.44173687 | 6.500936671 | 1.054169458 | 0.374889007 | 38.20124504 | 53.67344063 |
| AGA_89 | AGA | 3.822565026 | 5.760339071 | 1.619197137 | 0.626389974 | 51.64268653 | 49.43867431 |
| AGA_90 | AGA | 3.28812222 | 4.538477075 | 2.345217048 | 1.461039336 | 38.15132939 | 36.71231253 |
| AGA_91 | AGA | 4.142788038 | 4.281268263 | 1.296889563 | 1.746181877 | 45.5725299 | 53.43694575 |
| AGA_92 | AGA | 5.185119035 | 5.393535337 | 0.629694789 | 0.80772537 | 41.17874043 | 51.6615015 |
| AGA_93 | AGA | 4.671274995 | 3.943813434 | 0.899109463 | 2.206345403 | 48.83183861 | 52.1726307 |
| AGA_94 | AGA | 3.503408977 | 6.465167013 | 2.020111175 | 0.384300054 | 47.66929684 | 39.78601139 |
| AGA_95 | AGA | 4.638544741 | 5.943552097 | 0.919740598 | 0.551686277 | 53.30031955 | 41.35154768 |
| AGA_96 | AGA | 4.808253904 | 4.624659478 | 0.817669519 | 1.37631683 | 40.91986881 | 58.42522283 |
| AGA_97 | AGA | 3.792914051 | 3.629492377 | 1.652820002 | 2.743428012 | 42.84100052 | 49.68355877 |
| AGA_98 | AGA | 4.622980085 | 6.083097899 | 0.929717028 | 0.500823511 | 37.16989027 | 55.45200558 |
| AGA_99 | AGA | 4.546566975 | 4.908368124 | 0.980287407 | 1.130612046 | 41.44565392 | 50.22654701 |
| AGA_100 | AGA | 3.585623762 | 5.99025305 | 1.908209945 | 0.534113793 | 48.01840394 | 50.23804912 |
